# Role of extracellular matrix and microenvironment in regulation of tumor growth and LAR-mediated invasion in glioblastoma

**DOI:** 10.1371/journal.pone.0204865

**Published:** 2018-10-04

**Authors:** Yangjin Kim, Hyunji Kang, Gibin Powathil, Hyeongi Kim, Dumitru Trucu, Wanho Lee, Sean Lawler, Mark Chaplain

**Affiliations:** 1 Department of Mathematics, Konkuk University, Seoul, Republic of Korea; 2 Mathematical Biosciences Institute, Ohio State University, Columbus, Ohio, United States of America; 3 Molecular Imaging Research Center, Korea Institute of Radiological and Medical Sciences, Seoul, Republic of Korea; 4 Department of Mathematics, Swansea University, Swansea, United Kingdom; 5 Division of Mathematics, University of Dundee, Dundee, United Kingdom; 6 National Institute for Mathematical Sciences, Daejeon, Republic of Korea; 7 Department of neurosurgery, Brigham and Women’s Hospital & Harvard Medical School, Boston, Massachusetts, United States of America; 8 School of Mathematics and Statistics, Mathematical Institute, University of St Andrews, St Andrews, United Kingdom; University of California Irvine, UNITED STATES

## Abstract

The cellular dispersion and therapeutic control of glioblastoma, the most aggressive type of primary brain cancer, depends critically on the migration patterns after surgery and intracellular responses of the individual cancer cells in response to external biochemical cues in the microenvironment. Recent studies have shown that miR-451 regulates downstream molecules including AMPK/CAB39/MARK and mTOR to determine the balance between rapid proliferation and invasion in response to metabolic stress in the harsh tumor microenvironment. Surgical removal of the main tumor is inevitably followed by recurrence of the tumor due to inaccessibility of dispersed tumor cells in normal brain tissue. In order to address this complex process of cell proliferation and invasion and its response to conventional treatment, we propose a mathematical model that analyzes the intracellular dynamics of the miR-451-AMPK- mTOR-cell cycle signaling pathway within a cell. The model identifies a key mechanism underlying the molecular switches between proliferative phase and migratory phase in response to metabolic stress in response to fluctuating glucose levels. We show how up- or down-regulation of components in these pathways affects the key cellular decision to infiltrate or proliferate in a complex microenvironment in the absence and presence of time delays and stochastic noise. Glycosylated chondroitin sulfate proteoglycans (CSPGs), a major component of the extracellular matrix (ECM) in the brain, contribute to the physical structure of the local brain microenvironment but also induce or inhibit glioma invasion by regulating the dynamics of the CSPG receptor LAR as well as the spatiotemporal activation status of resident astrocytes and tumor-associated microglia. Using a multi-scale mathematical model, we investigate a CSPG-induced switch between invasive and non-invasive tumors through the coordination of ECM-cell adhesion and dynamic changes in stromal cells. We show that the CSPG-rich microenvironment is associated with non-invasive tumor lesions through LAR-CSGAG binding while the absence of glycosylated CSPGs induce the critical glioma invasion. We illustrate how high molecular weight CSPGs can regulate the exodus of local reactive astrocytes from the main tumor lesion, leading to encapsulation of non-invasive tumor and inhibition of tumor invasion. These different CSPG conditions also change the spatial profiles of ramified and activated microglia. The complex distribution of CSPGs in the tumor microenvironment can determine the nonlinear invasion behaviors of glioma cells, which suggests the need for careful therapeutic strategies.

## Introduction

Glioblastoma multiforme (GBM) is the most aggressive form of primary brain tumor and is characterized by rapid proliferation and aggressive invasion [1]. Poor clinical outcomes of glioblastoma are due to aggressive brain infiltration, driven in part by microRNA-mediated alterations in protein levels [2], leading to inevitable recurrence after surgery [3]. Conventional treatment methods such as surgery, primary treatment method, radiotherapy and chemotherapy have not proven to be effective [4] for this aggressive disease with a median survival time of approximately 15 months from the time of diagnosis [5–7]. In particular, invasive GBM cells, described as “*guerrilla-like warriors*”, can escape surgery and are protected behind the blood-brain barrier (BBB) and survive biochemical attacks from chemotherapy [8, 9]. Innovative new therapeutic approaches to block these invasive cells are needed in order to improve clinical outcome [10].

In the tumor microenvironment, cancer cells need to adapt to biochemical challenges including acidity, hypoxia, and limited nutrient availability [1]. In order to sustain rapid growth, cancerous cells modify their metabolic activity by increasing glycolysis even in the presence of oxygen, which requires high levels of glucose uptake, known as the *Warburg effect* [11, 12]. Differentiated cells favor oxidative phosphorylation via the tricarboxylic acid (TCA), or Krebs cycle, the major energy producing mechanism, which is very efficient in terms of ATP production. However, tumor cells adopt the seemingly inefficient process of aerobic glycolysis [13], which leads to consumption of large amounts of glucose and production of lactic acid [12]. Aerobic glycolysis [14] may provide tumor cells with the advantage of reducing the heavy dependency on oxygen for energy especially in the hypoxic tumor microenvironment, increasing a chance for longer survival and also promotes tumor growth by shuttling metabolites into biosynthetic pathways rather than ATP synthesis [12, 14]. Adequate cellular responses to glucose withdrawal are critical for glioma cell survival in the hostile microenvironment where glucose levels may fluctuate. Under metabolic stress, cells activate the 5′-adenosine monophosphate activated protein kinase (AMPK) pathway, the master cellular sensor of energy availability [15], in order to promote glucose uptake and to conserve energy [15], avoiding cell death.

**Fig 1:**
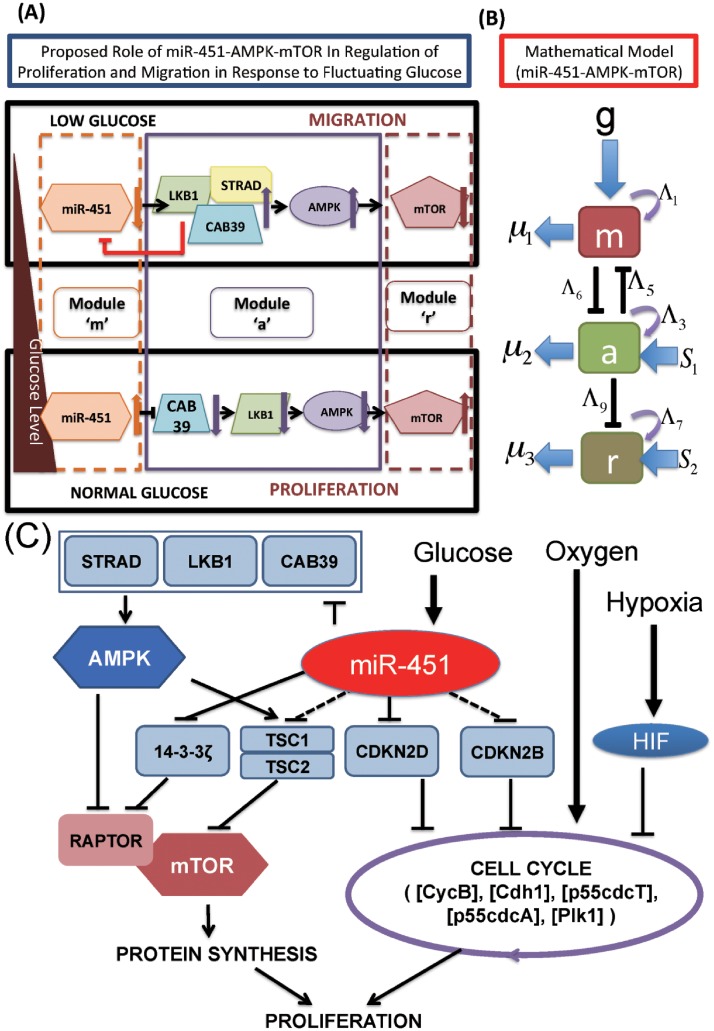
Proposed models of the miR-451-AMPK-mTOR-cell cycle signaling pathway. (A) Proposed role of miR-451 in the regulation of LKB1/AMPK-mTOR signaling in response to high and low glucose levels. miR-451 levels determine glioma cell migration or proliferation in response to glucose (triangle on the left) via the AMPK-mTOR network [1]. Glucose withdrawal reduces miR-451 levels, resulting in up-regulation of AMPK activity and down-regulation of mTOR. This leads to reduced cell proliferation and enhanced cell motility. Normal glucose levels up-regulate miR-451, which leads to down-regulation of AMPK activity. In turn, down-regulated AMPK levels induce up-regulation of mTOR, which leads to increased proliferation and decreased cell migration. (B) Schematic components of miR-451, CAB39/LKB1/AMPK complex, and mTOR are represented by modules ‘*M*’ (dotted box on the left), ‘*A*’ (box with solid line in the middle), and ‘*R*’ (dotted box on the right), respectively, in our theoretical framework. (C) Detailed schematic of cellular decision of cell proliferation and migration in glioblastoma via signaling networks including miR-451, AMPK, mTOR, and players in the cell cycle module (CycB, Cdh1, p55cdcT, p55cdcA, Plk1) [22].

miRNAs are approximately 22 nucleotide single-stranded non-coding RNAs that play a significant role in regulation of gene expression [16] and aberrant expression of microRNAs may suppress or promote malignant features of cancer depending on their context [2, 17]. Dysregulation of microRNA expression has been associated with oncogenic and tumor suppressor activities [18, 19] in several types of cancer, including GBM [20, 21]. Godlewski *et al.* [1, 22] identified the functional importance of miR-451 which targets the AMPK complex (LKB1/CAB39/STRAD/AMPK/MARK) and regulates cell fate in response to fluctuating glucose levels. (i) normal glucose levels induce up-regulation of miR-451 and down-regulation of AMPK complex, which induces elevated proliferation and decreased cell polarity/migration and (ii) glucose withdrawal leads to down-regulation of miR-451 and up-regulation of AMPK activity, which in turn induces increased cell polarity/migration and reduced cell proliferation. See Fig 1 for a schematic summary of miR-451-AMPK-mTOR core control system [1, 22].

Chondroitin sulfate proteoglycans (CSPGs), major components of the brain extracellular matrix (ECM) in the brain [23], are reported to play a pivotal role in inhibiting axon growth and regeneration process in scars after CNS damage [24]. CSPGs are also known to serve as biophysical barriers, preventing brain cells from migrating across the boundary of two adjacent structures during CNS development [25–32]. In CNS tumors, CSPGs may provide a structural foundation and guidance for tumor invasion [33] as well as a physical barrier for cell infiltration like other ECMs. Not surprisingly, this heavy chain of CSPGs has been associated with tumor growth, penetration into neighboring tissues, and angiogenesis [34]. The accumulating interstitial pressure and severe tortuosity in the extracellular space within the tumor [1] due to the high concentration of CSPG ECM are a severe limiting factor for efficient delivery of large therapeutic drugs [35]. Therefore, lowering the CSPG levels by an digestive enzyme was suggested as a way of promoting intratumoral transport of larger therapeutic compounds, making this ECM a potential target for adjuvant therapy [36, 37]. For example, tumor growth and invasion was reduced when these ECM molecules were digested by blocking antibodies against versican [36] or manipulating RNA against phosphacan [38]. CSPG degradation by classical proteases such as MMP-1 and MMP-8 also results in an increase in diffusion and hydraulic conductivity in solid tumors [39]. In oncolytic virus therapy, the diffusion-limiting properties of CSPGs are blamed for low therapeutic anti-tumor efficacy due to the inefficient intratumoral spread of oncolytic viruses within the compact glioma [40, 41]. The CSPG receptor LAR was shown to be involved in promoting or inhibiting tumor invasion [33, 42]. In recent work [42], CSPGs were suggested to be a biochemical indicator of non-invasive tumor and diffusely infiltrating tumor. In the study, they found that (i) a CSPG-rich microenvironment was associated with a strong cell-ECM adhesion and stimulated resident astrocytes to actively escape the CSPG-dense lesions, leading to the self-contained non-invasive tumor surrounded by astrocytes. (ii) Glycosylated CSPGs were not found in invasive GBM [42] and no collective migration of astrocytes was observed. (iii) These CSPG conditions also changed the profile of ramified and activated microglia within and outside the tumor. (iv) CSPG-induced cell-ECM bonding was mediated by its receptor LAR and LAR-CSGAG complex [42]. Integrins usually mediate the cell-matrix interactions by tethering the cell to its surrounding ECM and activating intracellular signalling cascades [43, 44].

Chondroitinase ABC I (Chase-ABC) is an enzyme that can eliminate Chondroitin sulfate glycosoamino glycans from proteoglycans without harmful effects *in vivo* [40] and has been widely tested for its effect on neuronal regeneration after CNS injury. This bacterial enzyme is known for its ‘loosening’ effect on the ECM scaffold [24, 45] and studied for enhancement of regeneration of injured axonal tracts. Chase-ABC was also suggested as an attractive target to enhance oncolytic virus (OV) spread in *in vivo* [40] and *in silico* [41] experiments and its anti-tumor effect has been proposed in Phase I/II trials for patients treated in Asia [46].

A glioma interacts with its microenvironment such as stromal cells (astrocytes, neurons, microglia, macrophages), ECM, vessels, and chemokines/cytokines by direct and indirect contacts in the tumor-stroma network [42, 43, 47, 48]. M1 and M2 types of microglia were shown to promote glioma cell invasion by exchanging signaling molecules such as CSF-1, EGF and TGF-*β* [49–52]. Astrocytes also play a significant role in the progression, aggression, and angiogenesis of CNS tumors [53, 54]. Mutual crosstalk between glioma cells and astrocytes plays a central role in cell infiltration through blood vessels through regulation of H^+^/Ca^2+^-channels [55] and is also responsible for resistance to therapy [53]. Resident astrocytes show reactive response with upregulation of glial fibrillary acidic protein (GFAP) and MMPs in a co-culture with glioma cells U87 [56]. These complex biomechanical interactions may deter tumor cell infiltration [57, 58]. However, the fundamental mechanism of formation of these two distinct (invasive vs non-invasive) gliomas is poorly understood.

Previously, Kim *et al.* developed a miR-451-AMPK core control model [59] and therapeutic anti-invasion strategies using localization of migratory glioma cells and dynamics of this critical signaling pathway [60, 61]. Mathematical models developed by Kim *et al.* [62, 63] and other research groups [64–68] showed that the go-or-grow pattern of glioma cells can be determined by key regulatory factors including cell-ECM interaction, chemotaxis toward nutrients (glucose, oxygen), and cell-cell adhesion [69]. Powathil *et al.* [70] developed a multi-scale model, based on a classical cell cycle model by Tyson and Novak [71, 72], to investigate the effects of hypoxia on the cell cycle and tumor growth. Lee *et al.* [73] developed an IBM-based, multi-scale model in order to investigate the role of myosin II in regulation of deformation of the membrane and nucleus that is necessary for glioma cell infiltration through narrow intercellular gaps between normal glial cells. Recently, Kim *et al.* [49] modeled and analyzed the biochemical interaction between a glioma and M1/M2 microglia for their promoting role of glioma invasion. A mathematical model developed in [41] showed that CSPGs can inhibit OV spread as a physical barrier, decreasing anti-tumor efficacy of OV therapies. However, how the glioma cell invasion is regulated by the miR-451-AMPK-mTOR-cell cycle signaling network and CSPG-mediated remodeling of microenvironment (microglia and astrocytes) in the brain is poorly understood.

In this paper, we develop and analyze a mathematical model that explores simultaneously the interlinked action of: (1) the miR-451-AMPK-mTOR-cell cycle signaling network, (2) LAR-CSGAG receptor dynamics of cell-ECM adhesion, and (3) a complex biomechanical hybrid processes involving microglia and astrocytes in GBM. This model will be then used to investigate (i) how up- or down-regulation of these pathways affect cell proliferation and migration in the absence and presence of time delays and stochastic noise. (ii) how the LAR-mediated ECM-cell adhesion is regulated, and (iii) how stromal cells are activated in tumor microenvironment and provide a feedback to the tumor invasion.

## Materials and methods

**Fig 2:**
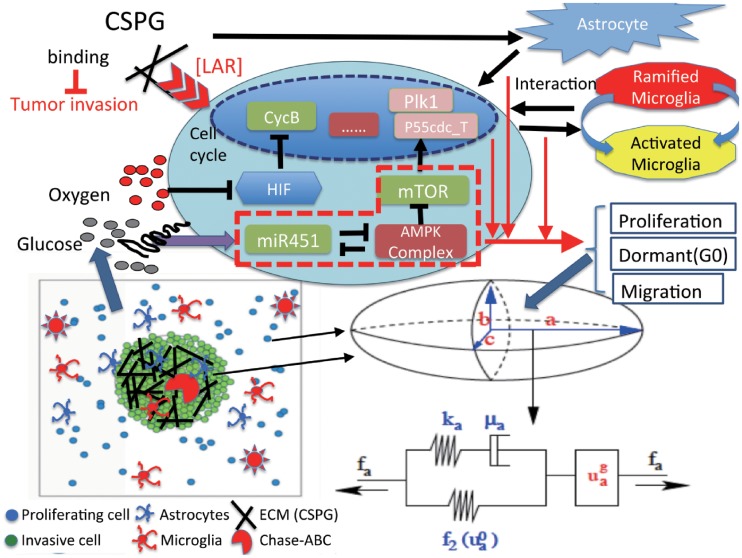
A schematic of the hybrid model. (Top) Intracellular dynamics of miR451-AMPK-mTOR and cell-cycle system in response to the diffusible molecules (glucose, oxygen) at a cell site. A heavy chain of the ECM component CSPG binds to its receptor, LAR, on a tumor cell, forming a biochemical interaction between ECM and the cell. The CSPG-rich tumor microenvironment also affect the movement of astrocytes and activation of ramified microglia. Those variables in the core system and LAR-CSGAG receptor dynamics, and spatial distribution of astrocytes and microglia determine the cell fate, proliferation, dormant stage (*G*_0_ phase) or critical infiltration of a glioma cell. (Bottom, Left) Model domain: A tumor cell (green) on the surface of tumor mass is activated to become a motile one (blue) via regulation of the intracellular dynamics, cell-matrix interaction, crosstalk with astrocytes (blue) and microglia (red). CSPGs (black bar) can be degraded via Chase-ABC (red). (Bottom, Right) Changes in the length of the *a*-axis of a tumor cell under a given force (*f*_*a*_; arrow) consist of two components: (i) the passive change in a Maxwell element in parallel with a non-linear spring (ii) the change due to the growth ($u_{a}^{g}$). The growth component depends on the nutrient-induced cell cycle and the force (*f*_*a*_).

Consider brain tissue, Ω = [0, *L*] × [0, *L*], with a tumor initially occupying a sphere, and astrocytes and ramified/activated microglia in the tumor microenvironment. A tumor cell either proliferates or migrates under certain biochemical conditions of miR-451-AMPK-mTOR-cell cycle activation and biomechanical binding to ECM from LAR-CSGAG regulation in response to nutrients (oxygen and glucose) and CSPG ECM according to the reaction-diffusion model. While mechanical movement of the tumor cell, astrocytes, and microglia is governed by the cell-based mechanical model, their migration direction is influenced by random motility and chemotaxis. On the other hand, the dynamics in the reaction-diffusion model depend on individual-cell components. A schematic of the hybrid model is shown in Fig 2. The multi-scale hybrid model contains the following components: (1) intracellular signaling pathway of a tumor cell (miR-451-AMPK-mTOR-cell cycle, LAR-CSGAG), (2) cell-based mechanical model (tumor cell, astrocytes, microglia), (3) reaction-diffusion model of extracellular biochemical players (oxygen, glucose, CSPG, Chase-ABC). In the next section, we introduce an intracellular model of cell proliferation and migration via miR-451-AMPK-mTOR-cell cycle.

### Intracellular dynamics

In this section, we introduce an intracellular model of cell proliferation and migration. We first introduce a miR-451-AMPK-mTOR signaling network.

#### miR-451-AMPK-mTOR core control

Let the variables *M*, *A* and *R* be activities of miR-451, AMPK complex, and mTOR, respectively. Based on the phenomenological network in Fig 1A, we obtain the following dimensionless model

$$\frac{dM}{dt}=\lambda_{g}G+\frac{\lambda_{1}\lambda_{2}^{2}}{\lambda_{2}^{2}+\alpha A^{2}}-M,$$ (1)

$$\epsilon_{1}\frac{dA}{dt}=S_{1}+\frac{\lambda_{3}\lambda_{4}^{2}}{\lambda_{4}^{2}+\beta M^{2}}-A,$$ (2)

$$\epsilon_{2}\frac{dR}{dt}=S_{2}+\frac{\lambda_{5}\lambda_{6}^{2}}{\lambda_{6}^{2}+\gamma A^{2}}-R.$$ (3)

See S1 Appendix for the derivation, nondimensionalization, parameter estimation of the model. Table 1 summarizes the definition and values of all the essential parameters in the core control system (1)–(3). The differential Eqs (1)–(3) are computationally integrated using a routine ode45 in MATLAB (The Mathworks Inc.) for simulations of the core control system model.

**Table 1:** Parameters used in the miR-451-AMPK-mTOR model.

Par	Description	Value	Refs
*λ*_*g*_	glucose signaling rate	1.0	[59]
*λ*_1_	autocatalytic production rate of miR-451	4.0	[59]
*λ*_2_	Hill-type coefficient	1.0	[59]
*α*	Inhibition strength of miR-451 by AMPK complex	1.6	[59]
*th*_*M*_	Threshold of AMPK for proliferation/migration	2.0	[59]
*λ*_3_	autocatalytic production rate of AMPK	4.0	[59]
*λ*_4_	Hill-type coefficient	1.0	[59]
*β*	Inhibition strength of AMPK complex by miR-451	1.0	[59]
*S*_1_	Signaling source of AMPK	0.2	[59]
*ϵ*_1_	Scaling factor (slow dynamics) of AMPK complex	0.02	[19, 59, 74, 75]
*th*_*A*_	Threshold of AMPK for proliferation/migration switch	2.0	[59]
*λ*_5_	autocatalytic production rate of mTOR	4.0	Estimated
*λ*_6_	Hill-type coefficient of mTOR module	1.0	Estimated
*γ*	Inhibition strength of mTOR activity by AMPK	1.0	Estimated
*S*_2_	Signaling source of mTOR	1.2	Estimated
*ϵ*_2_	Scaling factor (slow dynamics) of mTOR	0.02	[19, 74, 75], estimated
*th*_*R*_	Threshold of mTOR for proliferation/migration switch	3.0	Estimated

#### Cell-cycle pathway

The intracellular cell-cycle dynamics are modeled using a very basic model originally developed by Tyson and Novak [71, 72] that includes the various interactions which are considered to be essential for cell-cycle regulation and control. The Tyson and Novak model includes the kinetics of the chemical processes within the cell; namely the production, destruction and the interactions of different molecules involved by considering their concentrations as a function of time. Using these kinetic relations they explain the transitions between two main steady states, G1 and S-G2-M of the cell-cycle, which is controlled by changes in cell mass. Recently, Powathil *et al.* [70] used the equivalent mammalian proteins stated in Tyson and Novak’s paper, namely the Cdk-cyclin B complex [CycB], the APC-Cdh1 complex [Cdh1], the active form of the p55cdc-APC complex [p55cdc_A_], the total p55cdc-APC complex [p55cdc_T_], the active form of Plk1 protein [Plk1] and the mass of the cell [mass] to include the intracellular cell-cycle dynamics in their hybrid model. They have also modified the equations by incorporating the effects of hypoxia.

In the following, we introduce a new miR-451-AMPK-mTOR-cell cycle model. In this model, a new switch will be considered for *quiescent* stage (G0-phase) to explore the dynamic transition between migration and proliferation phases of the cell. The newly introduced *pseudo*-mass ([*mass*]^*s*^) will control the normal cell cycle and quiescent state so that it will put the tumour cell into *G*_0_ phase or in a typical cell cycle based on environmental stimuli such as glucose. The basic idea is that (i) when mTOR is up-regulated (proliferative phase; up-regulated miR-451, down-regulated AMPK), [*mass*_*s*_] is equal to [*mass*], hence we are back to typical cell cycle; and (ii) when mTOR is down-regulated (migratory phase; down-regulated miR-451; up-regulated AMPK), the switch function (second term in the Eq (13)) gives us a high value (*ζ*) that results in high values of [*mass*_*s*_], leading this way to the *G*_0_ phase. Therefore, the whole system of governing equations is as follows

$$\begin{array}{cc} & \frac{dM}{dt}=\lambda_{g}G\left(x,t\right)+\frac{\lambda_{1}\lambda_{2}^{2}}{\lambda_{2}^{2}+\alpha A^{2}}-M, \end{array}$$ (4)

$$\begin{array}{cc} & \epsilon_{1}\frac{dA}{dt}=S_{1}+\frac{\lambda_{3}\lambda_{4}^{2}}{\lambda_{4}^{2}+\beta M^{2}}-A, \end{array}$$ (5)

$$\begin{array}{cc} & \epsilon_{2}\frac{dR}{dt}=S_{2}+\frac{\lambda_{5}\lambda_{6}^{2}}{\lambda_{6}^{2}+\gamma A^{2}}-R. \end{array}$$ (6)

$$\begin{array}{cc} & \frac{d[CycB]}{dt}=k_{1}-\left(k_{2}^{{}^{\prime }}+k_{2}^{{}^{\prime \prime }}\left[Cdh1\right]+\left[p27/p21\right]\left[HIF\right]\right)\left[CycB\right] \end{array}$$ (7)

$$\begin{array}{cc} & \frac{d[Cdh1]}{dt}=\frac{\left(k_{3}^{{}^{\prime }}+k_{3}^{{}^{\prime \prime }}\left[p55cdc_{\text{A}}\right]\right)\left(1-\left[Cdh1\right]\right)}{J_{3}+1-\left[Cdh1\right]}-\frac{k_{4}\left[mass_{s}\right]\left[CycB\right]\left[Cdh1\right]}{J_{4}+\left[Cdh1\right]}, \end{array}$$ (8)

$$\begin{array}{cc} & \frac{d[p55cdc_{\text{T}}]}{dt}=k_{5}^{{}^{\prime }}+k_{5}^{{}^{\prime \prime }}\frac{{\left(\left[CycB\right]\left[mass_{s}\right]\right)}^{n}}{J_{5}^{n}+{\left(\left[CycB\right]\left[mass_{s}\right]\right)}^{n}}-k_{6}\left[p55cdc_{\text{T}}\right], \end{array}$$ (9)

$$\begin{array}{cc} & \frac{d[p55cdc_{\text{A}}]}{dt}=\frac{k_{7}\left[Plk1\right]\left(\left[p55cdc_{\text{T}}\right]-\left[p55cdc_{\text{A}}\right]\right)}{J_{7}+\left[p55cdc_{\text{T}}\right]-\left[p55cdc_{\text{A}}\right]}-\frac{k_{8}\left[Mad\right]\left[p55cdc_{\text{A}}\right]}{J_{8}+\left[p55cdc_{\text{A}}\right]}-k_{6}\left[p55cdc_{\text{A}}\right], \end{array}$$ (10)

$$\begin{array}{cc} & \frac{d[Plk1]}{dt}=k_{9}\left[mass_{s}\right]\left[CycB\right]\left(1-\left[Plk1\right]\right)-k_{10}\left[Plk1\right], \end{array}$$ (11)

$$\begin{array}{cc} & \left[mass\right]=k_{V}V_{i}, \end{array}$$ (12)

$$\begin{array}{cc} & \left[mass_{s}\right]=\left[mass\right]+\frac{\zeta_{1}{\left(1/R\right)}^{n_{1}}}{K_{m}^{n_{1}}+{\left(1/R\right)}^{n_{1}}}, \end{array}$$ (13)

$$\begin{array}{cc} & \left[HIF\right]=\zeta_{2}\left(G\left(x,t\right)\right)\frac{{\left(1/K\left(x,t\right)\right)}^{n_{2}}}{K_{H}^{n_{2}}+{\left(1/K\left(x,t\right)\right)}^{n_{2}}}, \end{array}$$ (14)

where *ζ*_1_, *ζ*_2_ are switching parameters for [*mass*_*s*_] and [*HIF*], *K*_*m*_, *K*_*H*_ are the Hill-type function parameters, *n*_1_, *n*_2_ are the Hill-type power parameters, and *G*(***x***, *t*), *k*(***x***, *t*) are the levels of glucose and oxygen, respectively, at space **x** and time *t*. It has been shown that glucose deprivation down-regulates [*HIF*] ([*HIF*] = 0) [76, 77]. Following these biological observations [76, 77], we assume that the switching parameter of [*HIF*], *ζ*_2_, is glucose-dependent, *i.e.,*

$$\begin{array}{c} \zeta_{2}\left(G\left(x,t\right)\right)=\{\begin{array}{cc} 1 & \text{if}\;G\left(x,t\right)<th_{G}^{†}\\ 0 & \text{otherwise}, \end{array} \end{array}$$ (15)

where $th_{G}^{†}$ is the threshold of the glucose level for the [*HIF*]-switch.

Table 2 summarizes all the parameter values in the cell cycle module (7)–(15).

**Table 2:** Parameters used in the cell-cycle module: Dimensionless values were marked in *.

Par	Description	Value	Refs
cell cycle module			
*k*_1_	production rate of [*CycB*]	1.2 × 10^−1^ *h*^−1^	[70–72]
$k_{2}^{\prime }$	degradation rate of [*CycB*]	1.2 × 10^−1^ *h*^−1^	[70–72]
$k_{2}^{{}^{\prime \prime }}$	degradation rate of [*CycB*] by [*Cdh*1]	4.5 *h*^−1^	[70–72]
[p27/p21]	inhibition rate by [*HIF*1]	1.05 *h*^−1^	[70]
[CycB]_*th*_	threshold of [CycB] for cell division	1.0 × 10^−1^	[70–72]
$k_{3}^{\prime }$	activation rate of [*Cdh*1]	3.0 *h*^−1^	[70–72]
$k_{3}^{{}^{\prime \prime }}$	activation rate of [*Cdh*1] by [*p*55*cdc*_A_]	3 × 10^1^ *h*^−1^	[70–72]
*k*_4_	inactivation rate of [*Cdh*1] by [*CycB*]	1.05 × 10^2^ *h*^−1^	[70–72]
*J*_3_	Michaelis-Menton constant (activation)	*4.0 × 10^−2^	[70–72]
*J*_4_	Michaelis-Menton constant (inactivation)	*4.0 × 10^−2^	[70–72]
$k_{5}^{\prime }$	production rate of [*p*55*cdc*_T_]	1.5 × 10^−2^ *h*^−1^	[70–72]
$k_{5}^{{}^{\prime \prime }}$	transcription rate of [*p*55*cdc*_T_] by [*CycB*]	6.0 × 10^−1^ *h*^−1^	[70–72]
*k*_6_	degradation rate of [*p*55*cdc*_T_]	3.0 × 10^−1^ *h*^−1^	[70–72]
*J*_5_	dissociation constant of [*p*55*cdc*_T_]	*3.0 × 10^−1^	[70–72]
*n*	Hill coefficient	*4	[70–72]
*k*_7_	activation rate of [*p*55*cdc*_A_] by [*Plk*1]	3.0 *h*^−1^	[70–72]
*k*_8_	inactivation rate of [*p*55*cdc*_A_] by [*Mad*]	1.5 *h*^−1^	[70–72]
*J*_7_	Michaelis-Menton constant (activation)	*1.0 × 10^−3^	[70–72]
*J*_8_	Michaelis-Menton constant (inactivation)	*1.0 × 10^−3^	[70–72]
[*Mad*]	concentration of [*Mad*]	*1.0	[70–72]
*k*_9_	activation rate of [*Plk*1] by [*CycB*]	3.0 × 10^−1^ *h*^−1^	[70–72]
*k*_10_	degradation rate of [*Plk*1]	6.0 × 10^−2^ *h*^−1^	[70–72]
*μ*^+^	Growth rate parameter in [*mass*]	3.0 × 10^−2^ *h*^−1^	[71, 72]
m_*_	Growth rate parameter in [*mass*]	10	[71, 72]
*ϵ*	small parameter for *μ*	*6.0 × 10^−3^	[70–72]
miR-451-AMPK-mTOR-cell cycle (ODE)			
*ζ*_1_	*G*_0_ switch parameter	*2.5	TW
*n*_1_	Hill-type parameter in *G*_0_ switch	*10	TW
*K*_*m*_	Hill-type parameter in *G*_0_ switch	*0.5	TW
*ζ*_2_	[*HIF*] switch parameter	*1.0	TW
*n*_2_	Hill-type parameter in [*HIF*] switch	*10	TW
*K*_*H*_	Hill-type parameter in [*HIF*] switch	*10.0	TW
$th_{G}^{†}$	the threshold of glucose for [*HIF*] activation	*0.4	TW

### Dynamics of CSGAG-LAR receptor binding

In this work, we consider the following chemical reactions of [CS-GAG] and its receptor [LAR] on the glioma cell:

$$\begin{array}{c} \left[E\right]+\left[R\right]\overset{k_{on}}{\underset{k_{off}}{⇌}}\bar{E\cdot R}, \end{array}$$ (16)

where $\left[E\right],\left[R\right],\bar{E\cdot R}$ are concentrations of CSPG, its receptor LAR, and complex [CSPG] ⋅ [LAR], respectively, and *k*_*on*_ and *k*_*off*_ are reaction rate constants. We assume that the total number [*R*_*T*_] of receptor, sum of free and bound receptors, remains constant, *i.e.,* $\left[R_{T}\right]=\left[R\right]+\bar{E\cdot R}=\text{constant}$. Given the CSPG concentration *E*_*i*_ at the tumor cell site *i*, the level of the complex $\bar{E\cdot R}$ in (16) satisfies the following differential equation:

$$\frac{d[R]}{dt}=-k_{on}[E]\cdot [R]+k_{off}\bar{E\cdot R},$$ (17)

$$\begin{array}{ccc} & & \frac{d\bar{E\cdot R}}{dt}=k_{on}\left[E\right]\cdot \left[R\right]-k_{off}\bar{E\cdot R},=k_{on}\left[R_{T}\right]\left[E\right]-\left(k_{on}\left[E\right]+k_{off}\right)\bar{E\cdot R}, \end{array}$$ (18)

$$\begin{array}{ccc} & & \left[R_{T}\right]=\left[R\right]+\bar{E\cdot R}=\text{constant}. \end{array}$$ (19)

In the results section we analyze the dynamics of CSPG-LAR receptor binding. The steady state of the bound myosin concentration ($\left[\bar{E\cdot R}\right]$) in the Eq (18) is given by

$$\begin{array}{c} {\left[\bar{E\cdot R}\right]}^{s}=\frac{k_{1}\left[R_{T}\right]\left[E\right]}{k_{1}\left[E\right]+k_{-1}}=\left[R_{T}\right]\frac{\left[E\right]}{\left[E\right]+K_{D}} \end{array}$$ (20)

where $K_{D}=\frac{k_{off}}{k_{on}}$ is the ratio of the association rate (*k*_*on*_) to the dissociation rate (*k*_*off*_). Table 3 summarizes all the parameter values in the LAR receptor module (17)–(19).

**Table 3:** Parameters that are used in the [CSPG]-[LAR] receptor dynamics.

Par	Description	Value	Refs
**[CSPG]-[LAR] binding dynamics**			
E*	Reference value for CSPG	500 *μg*/*ml*	[1]
*k*_1_	Association rate	2.4 × 10^−4^ *nM*^−1^ *s*^−1^	[78–80]
*k*_−1_	Dissociation rate	7.1 × 10^−4^ *s*^−1^	[78–80]
[*R*_*T*_]	Total LAR receptor concentration	10 *nM*	[78–80]
**Active force generation from [CSPG]-[LAR] binding**			
*ϵ*_*i*_	Scaling factor	0.001*nM*	Estimated
*K*_*ER*_	Hill function coefficient	0.5	Estimated
*m*_1_	Hill function coefficient	2	Estimated
*S*_*ER*_	Hill function coefficient	0.2*nM*	Estimated

### Mechanical effects on tumor growth: The cell-based component

Cell-mechanics plays a significant role in regulation of tumor growth and invasion in the presence of tumor microenvironment [81]. Mechanical stresses [82], cell-ECM interaction [1], and the core signaling pathways are considered here to influence cell proliferation and migration in a phenomenologically-specified manner.

#### The forces acting on individual cells

The basic bio-mechanical part of individual cells is based on the mechanical models developed by Dallon and Othmer [83] and the hybrid model by Kim *et al.* [84]. The basic scheme for cell division under given stress conditions was developed in the hybrid model [84]. Further applications of the hybrid model with an intracellular dynamics to breast cancer with EGF-TGF-*β* signaling and glioblastoma with the miR-451-AMPK signaling can be found in [63, 85, 86]. The new aspects of tumor cells in the present paper are the core control system (miR-451-AMPK-mTOR-cell cycle) and cell-ECM adhesion system (LAR-CSGAG) for glioma cell proliferation and migration in the presence of biomechanical feedback from microglia and astrocytes. The forces on a cell in [83] include, (i) the traction forces acting on the substrate or neighboring cells, (ii) the dynamic drag forces that begin to occur as a migratory cell generates and breaks chemical bonds with neighboring cells, (iii) a static frictional force that arises when cells are firmly attached to the substrate or to each other, and (iv) a reactive force due to responding forces exerted by other cells on it. The total force on the *i*th cell is given by

$$\begin{array}{c} F_{i}=\sum_{j\in N_{i}^{a}}M_{j,i}+\sum_{j\in N_{i}^{a}}T_{j,i}+\sum_{j\in N_{i}^{d}}\mu_{ij}\left(v_{j}-v_{i}\right)+\sum_{j\in N_{i}^{s}}S_{j,i} \end{array}$$ (21)

where **T**_*i,j*_, **M**_*j,i*_, **S**_*j,i*_ are the traction force, reaction force, and static force on a cell *i*, respectively, $N_{i}^{a}$ is the neighbors of *i*, including the substrate, $N_{i}^{d}$ denotes the set of cells (which includes substrate and ECM) that interact with *i* via a frictional force, and $N_{i}^{s}$ denotes the set of cells that forms a static bond to cell *i*. See [83, 84] for a detailed discussion of all forces involved.

#### Cell growth and the rheology of the cytoplasm

There are various cell types involved in the system: proliferative glioma cells, motile glioma cells, ramified microglia, activated microglia, and astrocytes. These cells are treated as oriented ellipsoids and their cytoplasm considered as an incompressible, viscoelastic solid [83–85]. Evolution of these cells are based on force balance equation that describes the motion of these cells in response to chemotactic signals. The length of the *i*-th axis, *i* = **a**, **b**, **c**, of a cell is given by [83–85]

$$\begin{array}{ccc} & & u_{i}=u_{i}^{0}+u_{i}^{g}, \end{array}$$ (22)

$$\begin{array}{ccc} & & \frac{du_{i}^{0}}{dt}=(\frac{k_{i}}{\mu_{i}}\left[f_{i}\left(t\right)+\bar{p}-f_{2}\left(u_{i}^{0}\right)\right]+\frac{df_{i}}{dt})\times {\left(f_{2}^{\prime }\left(u_{i}^{0}\right)+k_{i}\right)}^{-1}, \end{array}$$ (23)

where *u*_*i*_ is the total change in the length of the *i*th axis, $u_{i}^{0}$ and $u_{i}^{g}$ are the changes in the length of the *i*th axis due to contributions from the intrinsic (passive) and additional growth elements, respectively, *f*_*i*_ is the magnitude of the force acting at each end, $\bar{p}$ is the pressure, *f*_2_ is the force from the nonlinear spring in parallel, *k*_*i*_ is the spring coefficient in the Maxwell element, *μ*_*i*_ is the viscous constant of the dashpot. See [83] for the specific form of the function *f*_2_ and how these equations are established in more detail. These equations are coupled with an equation of a constant volume under the assumption that the passive response is incompressible. The growth rate on the *i*-th axis is given by

$$\frac{du_{i}^{g}}{dt}=f\left(\sigma \right)\cdot P\left(\left[G_{0}\right]\right)$$ (24)

$$P\left(\left[G_{0}\right]\right)=\{\begin{array}{ll} 1 & \text{if}\;\text{normal}\;\text{cell}\;\text{cycle}\left(\left[G_{0}\right]=0\right)\\ 0 & \text{if}\;G_{0}\;\text{phase}\;\left(\left[G_{0}\right]=1\right) \end{array}$$ (25)

where *σ* is the force acting on the tumor cell and the function *P* determines a proliferation switch based on the cell cycle status (normal cell cycle or quiescent status ([*G*_0_])). The growth function *f*(*σ*) is defined so that cells do not grow if forces are too large, but can grow under sufficiently small tensile and compressive forces [84, 85].

#### Active force and equations of motion

A cell generates the active force for cell migration either as a collective migration or as a single cell motion by invoking complex bio-mechanical processes. These processes include tightly controlled signaling pathways [87, 88], control of tension, traction force generation on the adhesive binding sites [83], and feedback regulation of the actomyosin network [89, 90]. For example, the myosin II plays a significant role in glioma cell infiltration through the narrow intercellular gap between normal glial cells in tumor microenvironment by deforming both cell membrane and nucleus [91]. This specific role of myosin II and effect of its inhibitor such as Blebbistatin was investigated by Lee *et al.* [73]. In this work, we don’t consider a detailed process of myosin II-mediated nucleus deformation or collective cell migration, which are certainly key aspects in a critical stage of many cancers [90]. Instead, we take into account the cell-ECM adhesion on initiation of tumor cell invasion. Here, we assume that the traction force is generated for only the cells that are under the following circumstances: (i) without physical constraints, (ii) that receive the correct migratory signal, (iii) that are free of the strong LAR-CSGAG binding.

The traction force $T_{i}^{a}$ for a migratory infiltrating glioma cell *i* is given by

$$\begin{array}{c} T_{i}^{a}=\phi \left(\zeta_{i}\right)(\psi_{1}d_{r}+\psi_{2}\frac{\nabla G}{\sqrt{K_{G}+{\left|\nabla G\right|}^{2}}}+\psi_{3}\frac{\nabla C}{\sqrt{K_{C}+{\left|\nabla C\right|}^{2}}})\equiv \phi \left(\zeta_{i}\right)T_{i,g} \end{array}$$ (26)

where **d**_*r*_ is a unit vector indicating the moving direction from random motion, *G*, *C* are the concentrations of glucose and a chemoattractant, respectively (described below). Further, *ψ*_1_, *ψ*_2_, *ψ*_3_ are scaling factors of weight distribution favouring random movement, chemotactic movement toward glucose and directed movement toward other chemoattractants, respectively (*ψ*_1_, *ψ*_2_, *ψ*_3_ ∈ [0, 1]; *ψ*_1_+ *ψ*_2_+ *ψ*_3_ = 1). Finally, *ζ*_*i*_ is the unbinding strength in [CSPG]-[LAR] reaction at the *i*th cell site. More precisely, the unbinding strength to CSPGs (*ζ*_*i*_) at the cell site *i* is given by

$$\begin{array}{c} \zeta_{i}=\zeta_{i}(\left[\left(\bar{E\cdot R}\right]\right)=\frac{{\left[S_{ER}/\left(\left[\bar{E\cdot R}\right]+\epsilon_{i}\right)\right]}^{m_{1}}}{K_{ER}^{m_{1}}+{\left[S_{ER}/\left(\left[\bar{E\cdot R}\right]+\epsilon_{i}\right)\right]}^{m_{1}}} \end{array}$$ (27)

where $\left[\bar{E\cdot R}\right]$ is the [CS-GAG]-[LAR] levels, and $m_{1},K_{ER},S_{ER},\epsilon_{i}\;\left(m_{1}\in Z^{+},K_{ER}\in R^{+},\epsilon_{i}\in R^{+},\epsilon_{i}⪡1\right)$ are Hill-type coefficients. Then, the indicator function *ϕ*(*ζ*_*i*_) is given by

$$\begin{array}{c} \phi \left(A_{i},\zeta_{i}\right)=\{\begin{array}{cc} F_{0}\zeta_{i}\phi_{r}, & \;\text{if}\;\text{migration}\;\text{signal}\;\text{without}\;\text{physical}\;\text{constraints}\\ 0, & \text{otherwise}, \end{array} \end{array}$$ (28)

where *F*_0_ is the basal magnitude of the traction force ($0\leq |T_{i}^{a}|\leq F_{0}$) and *ϕ*_*r*_ is a random number between 0.8 and 1.2. We note that a small randomness in the magnitude of traction force is allowed. So, the traction force is completely turned off for glioma cells in the growth phase, adhesive cells with high affinity to CSPGs via [CS-GAG]-[LAR] binding, or cells under physical constraints such as cells inside the dense tumor core. A cell is considered to be under physical constraints when other cells are present in the migration direction (**T**_*i,g*_). For a more precise algorithm, let *j*, the index of a neighboring cell whose center position is in the closest direction to the migration direction (**T**_*i,g*_), be given by $j=\{k:max_{k\in N_{i}^{a}}\frac{x_{k}-x_{i}}{|x_{k}-x_{i}|}\cdot {\hat{T}}_{i}\}$ where ${\hat{T}}_{i}=\frac{T_{i,g}}{|T_{i,g}|}$, **x**_*k*_ is the location of the center of the *k*-th cell, $N_{i}^{a}=\{k:0<|x_{k}-x_{i}|\leq d_{n}\}$ is the cells in the neighborhood of the cell *i*. The cell *i* is under physical constraints if *j* > 0. For example, the traction force is completely turned off when the glioma cell is completely surrounded by neighboring cells in the tumor core [61] or its infiltration is blocked by a thick layer of astrocytes at the edge of the growing tumor [42].

The traction force for reactive astrocytes is given by $S_{a}*\frac{E^{m_{2}}}{K_{E}^{m_{2}}+E^{m_{2}}}(\psi_{1}^{a}d_{r}+\psi_{2}^{a}\frac{\nabla G}{\sqrt{K_{G}+{\left|\nabla G\right|}^{2}}})$ where *S*_*a*_ is the intrinsic force of astrocytes, *E*, *G* are the concentration of CSPGs and nutrients (glucose,oxygen), respectively, *K*_*E*_, *m* are the Hill-type constants of migration activation, $\psi_{1}^{a},\psi_{2}^{a}$ are scaling factors of weight distribution favoring random motion, nutrient (oxygen and glucose), respectively ($\psi_{1}^{a},\psi_{2}^{a}\in \left[0,1\right]$; $\psi_{1}^{a}+\psi_{2}^{a}=1$).

The force balance on cells in a migratory phase requires the following specific forces: the force of reaction ($T_{i}^{a,*}=-T_{i}^{a}$) to the given traction force $T_{i}^{a}$, adhesive forces between two cells (**A**_*i,j*_), the drag effect due to the surrounding fluid acting on the cell, internal forces (**R**_*i,j*_), and the passive reactive force from deformation of the cell from cell-substrate ($R_{0,i}^{*}$) and cell-cell ($R_{j,i}^{*}$) interactions. In particular, the cell-cell adhesion strength between tumor cells is very high when $\left[\bar{E\cdot R}\right]$ is high but is very low when $\left[\bar{E\cdot R}\right]$ is low. By using Eqs (21)–(28) and neglecting acceleration, the governing equation of motion for *i*-th cell is given by,

$$\begin{array}{l} A_{if}\mu_{f}v_{i}+A_{is}\mu_{s}v_{i}+\mu_{cell}\sum_{j\in N_{i}}A_{ij}(v_{i}-v_{j})+\\ \;+\frac{A}{6\pi r_{ib}}(T_{i}^{a,*}+R_{0,i}^{*}+\sum_{j\in N_{i}}A_{i,j}+\sum_{j\in N_{i}}R_{j,i}+\sum_{j\in N_{i}}R_{j,i}^{*})=0, \end{array}$$ (29)

where **V**_*i*_ is the cell velocity, $N_{i}$ is the neighboring cells of the cell *i*, and *μ*_*cell*_ (resp., *μ*_*s*_, *μ*_*f*_) is the degree of the cell-cell adhesion (resp., between the substrate and the cells, and the fluid viscosity). Further, *r*_*ib*_ = *u*_*b*_+ *b*_0_, and *A*_*ij*_, *A*_*if*_ = *A*_*if*_, *A*_*is*_ are the contact area between cell *i* and cell *j*, cell *i* and the interstitial fluid or matrix, and cell *i* and the substrate, respectively. Finally, *A* is the total area of an undeformed cell. For more details see Dallon and Othmer [83]. Parameters in the cell-based component are listed in Table 4.

**Table 4:** Parameters for the cell-based component of the model. TW = this work. *dimensionless value.

Par	Description	Value	Refs.
Adhesion parameters			
*μ*_*cell*_	cell-cell adhesiveness	27.0 dyn s/cm	[83]
*μ*_*s*_	cell-substrate adhesiveness	27.0 dyn s/cm	[83]
*μ*_*f*_	the fluid viscosity	2.7 dyn s/cm	[83]
Rheological parameters			
*c*^+^	Growth function parameter	1.016089 × 10^−7^ mm/(min.nN)	[84], TW
*σ*^+^	Growth function parameter	800 nN	[84]
*σ*^−^	Growth function parameter	-4 nN	[84]
*k*_*a*_	Standard solid parameter in cell	163.8 dyn/cm	[83, 84]
*k*_2_	Standard solid parameter in cell	147.5 dyn/cm,	[83, 84]
*μ*_*a*_	Standard solid parameter in cell	123 dyn min/cm	[83, 84]
Active force parameters			
*ψ*_1_	Weight for random motility (*ψ*_1_+ *ψ*_2_+ *ψ*_3_ = 1)	0-1.0	Estimated
*ψ*_2_	Weight for glucose gradient (*ψ*_1_+ *ψ*_2_+ *ψ*_3_ = 1)	0-1.0	Estimated
*ψ*_3_	Weight for chemoattractant gradient (*ψ*_1_+ *ψ*_2_+ *ψ*_3_ = 1)	0-1.0	Estimated
*F*_0_	Maximal active force magnitude	64 *nN*	[83]
*ϕ*_*r*_	Random factor for basal active force	0.8-1.2	[83]
*K*_*G*_	Active force scaler for the glucose gradient	1.0*	TW
*K*_*C*_	Active force scaler for the chemoattractant gradient	1.0*	TW

#### Activation of microglia

Microglia have two states: ramified or activated states. One state transits to another state based on CSPG concentration and tumor density. We define the transition probability density function at the microglia site **x**_*i*_

$$P_{A}(x_{i})=\frac{1}{\left|B_{S_{R}}(x_{i})\right|}\int_{B_{S_{R}}(x_{i})}E(y,t)dy$$ (30)

where *E*(**y**, *t*) is a CSPG density within the sensing radius *S*_*R*_ [62]. We set *S*_*R*_ = 20 − 100*μm*.

### Dynamics of biochemical players

The macroscopic dynamics of concentrations of relevant biochemical players (oxygen, glucose, CSPG, Chase-ABC) is modeled using a suitable system of partial differential equations. Chase-ABC, an enzyme, was reported to degrade CSPGs [41], reducing the inhibitory properties of CSPGs [92, 93]. Let *k*(***x***, *t*), *G*(***x***, *t*), *E*(***x***, *t*), *A*(***x***, *t*) denote the concentration of oxygen, glucose, CSPG ECM, and Chase-ABC at spatial position **x** and time *t*, respectively. Their rate of change can be expressed as

$$\frac{\partial K}{\partial t}=\underset{\text{Diffusion}}{\underset{︸}{\nabla \cdot (D_{K}(x)\nabla K)}}+\underset{\text{Supply}}{\underset{︸}{r_{K}I_{B}(x)}}-\underset{\text{Consumption}}{\underset{︸}{l_{c}^{K}I_{C}(x)}}-\underset{\text{Decay}}{\underset{︸}{\mu_{K}K}}$$ (31)

$$\begin{array}{ccc} & & \frac{\partial G}{\partial t}=\underset{\text{Diffusion}}{\underset{︸}{\nabla \cdot (D_{G}\left(x\right)\nabla G)}}+\underset{\text{Supply}}{\underset{︸}{r_{G}I_{B}\left(x\right)}}-\underset{\text{Consumption}}{\underset{︸}{l_{c}^{G}I_{C}\left(x\right)}}-\underset{\text{Decay}}{\underset{︸}{\mu_{G}G}} \end{array}$$ (32)

$$\begin{array}{ccc} & & \frac{\partial E}{\partial t}=-\underset{\text{Removal}\;\text{by}\;\text{Chase-ABC}}{\underset{︸}{\frac{\mu_{E}\;E\;A}{K_{A}+A}}}, \end{array}$$ (33)

$$\begin{array}{ccc} & & \frac{\partial A}{\partial t}=\underset{\text{Diffusion}}{\underset{︸}{\nabla \cdot (D_{A}\nabla A)}}-\underset{\text{Degradation}}{\underset{︸}{\mu_{EA}EA}}-\underset{\text{Natural}\;\text{decay}}{\underset{︸}{\mu_{A}A}} \end{array}$$ (34)

where *D_K_*(**x**), *D_G_*(**x**), *D_A_*(**x**) are the diffusion coefficients of oxygen, glucose, and Chase-ABC, respectively, *μ*_*K*_, *μ*_*G*_, *μ*_*A*_ are the natural decay rates of oxygen, glucose, and Chase-ABC, respectively, *μ*_*E*_ is the degradation rate of CSPG ECM by Chase-ABC, *K*_*A*_ is a Hill-type parameter, *μ*_*EA*_ is the consumption rate of Chase-ABC in the process of CSPG degradation, *r*_*K*_, *r*_*G*_ are the supply rates of oxygen and glucose at blood sites, respectively, $l_{c}^{K},l_{c}^{G}$ are consumption rates of oxygen and glucose at cell sites, respectively. Here, *I*_*C*_(⋅) is an indicator function on the cell sites.

$$\begin{array}{c} I_{C}\left(x\right)=\{\begin{array}{cc} 1 & \text{cells}\\ 0 & \text{otherwise}. \end{array} \end{array}$$ (35)

Similarly, *I*_*B*_(⋅) is an indicator function on the blood sites *B*,

$$\begin{array}{c} I_{B}\left(x\right)=\{\begin{array}{cc} 1 & \text{blood}\;\text{vessels}\\ 0 & \text{otherwise}. \end{array} \end{array}$$ (36)

Table 5 summarizes all the parameter values in the reaction-diffusion module (31)–(36).

**Table 5:** Parameters that are used in the reaction-diffusion equations.

Par	Description	Value	Refs
*D*_*K*_	Diffusion coefficient of oxygen	2.0 × 10^−5^ *cm*^2^/*s*	[94–96]
*D*_*G*_	Diffusion coefficient of glucose	6.7 × 10^−7^ *cm*^2^/*s*	[62, 97, 98]
*D*_*A*_	Diffusion coefficient of Chase-ABC	1.08 × 10^−9^ *cm*^2^/*s*	[41, 99]
*r*_*K*_	Oxygen supply rate from blood	6.35 × 10^−4^ *g*/(*cm*^3^.*s*)	Estimated
*r*_*G*_	Glucose supply rate from blood	1.4 × 10^−3^ *g*/(*cm*^3^.*s*)	Estimated
$l_{c}^{K}$	Oxygen consumption rate by tumor	0.8 *pg*/*cell*/*min*	Estimated
$l_{c}^{G}$	Glucose consumption rate by tumor	0.8 *pg*/*cell*/*min*	[100, 101]
*μ*_*K*_	Removal rate of oxygen in brain tissue	2.0 × 10^−5^ *s*^−1^	[102]
*μ*_*G*_	Removal rate of glucose in brain tissue	0.0034 *min*^−1^	Estimated
*μ*_*E*_	Chase-ABC-mediated CSPG degradation rate	1.19 × 10^−2^ *s*^−1^	[40, 41]
*K*_*A*_	Hill-type parameter for CSPG degradation	50 *mU*/*ml*	[24, 41, 45, 103]
*μ*_*EA*_	Reaction rate of Chase-ABC for CSPG degradation	1.15 × 10^4^ *mm*^3^/(*g*.*h*)	[41]
*μ*_*A*_	Natural decay rate of Chase	3.0 × 10^−3^ (1/*h*)	[24, 41]

## Results

### Dynamics of the core control system

**Fig 3:**
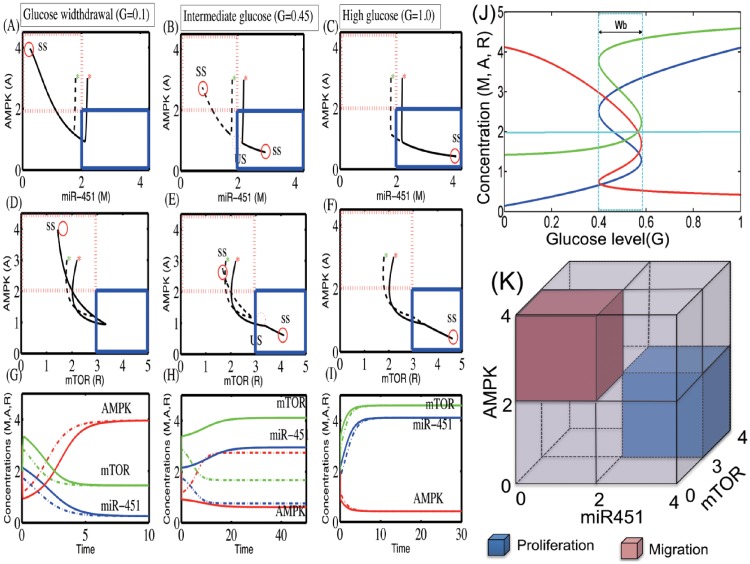
Effect of glucose on regulation of the core control system. (A-F) Trajectories of concentrations of core control system variables (miR-451, AMPK, mTOR) in miR-451-AMPK-mTOR space, in response to low (*G* = 0.1, (A)), intermediate (*G* = 0.45, (B)), and high (*G* = 1.0, (C)) glucose levels. (G-I) Time courses of miR-451 (*M*), AMPK complex (*A*), and mTOR (*R*) in response to low (C), intermediate (F) and high (I) glucose levels. Initial conditions: *M*(0) = 2.2, *A*(0) = 3.0, *R*(0) = 2.2 (solid lines), *M*(0) = 1.8, *A*(0) = 3.0, *R*(0) = 1.8 (dotted lines). *Dotted box = migratory zone ($T_{m}$), Solid box = proliferative zone ($T_{p}$) in (A-F). (J) Steady-state bifurcation diagrams (adapted from [61]). High and low glucose levels (*G*) provide an on-off switch of miR-451 over-expression and determine the dichotomous behavior: cell proliferation or migration. Y-axis = steady state (SS) of miR-451 (in (A)), AMPK (in (B)), and mTOR (in (C)). *W*_*b*_ = $\left[b_{m}^{w},b_{M}^{w}\right]$ = a window of bi-stability. (K) Characterization of proliferation and migration of glioma cells (adapted from [61]). The proliferative region is defined as the region where the miR-451 level is above a threshold, *th*_*M*_ (*M* > *th*_*M*_), the AMPK level is below a threshold, *th*_*A*_ (*A* > *th*_*A*_), and the mTOR level is above a threshold, *th*_*R*_ (*R* > *th*_*R*_), while the levels of miR-451, AMPK, and mTOR in the migratory region satisfies *M* < *th*_*M*_, *A* > *th*_*A*_, *R* < *th*_*R*_. We set *th*_*M*_ = 2.0, *th*_*A*_ = 2.0, *th*_*R*_ = 3.0.

We recall (Fig 1) that low levels of miR-451 (up-regulated AMPK complex and down-regulated mTOR) induce reduced cell proliferation and increased cell motility while over expression of miR-451 (down-regulation of AMPK complex and up-regulation of mTOR) leads to elevated cell proliferation and reduced migration in the experimental setting [1, 22]. In order to take into account the effect of glucose conditions in our model on phenotypic changes (proliferative versus migratory), we first test how the glucose level (*G*) affects the levels of key players (*M*, *A*, *R*) in our core control system. In Fig 3 we illustrate three different patterns of the steady state (SS; circles in Fig 3A–3F) of the core control system in response to low (*G* = 0.1; Fig 3A and 3D), intermediate (*G* = 0.45; Fig 3B and 3E), and high (*G* = 1.0; Fig 3C and 3F) levels of glucose in the *M*-*A* and *R*-*A* phase diagrams. By taking the thresholds, *th*_*M*_ (= 2.0) of miR-451 levels, *th*_*A*_ (= 2.0) of AMPK complex, and *th*_*R*_ (= 3.0) of mTOR, we shall define the migratory region $T_{m}$ (red dotted boxes in Fig 3A–3F) by

$$\begin{array}{c} T_{m}=\left\{\left(M,A,R\right)\in R^{2}:M<th_{M},\;A>th_{A},\;R<th_{R}\right\} \end{array}$$

and the proliferative region $T_{p}$ (blue dotted boxes in Fig 3A–3F) by

$$\begin{array}{c} T_{p}=\left\{\left(M,A,R\right)\in R^{2}:M>th_{M},\;A<th_{A},\;R>th_{R}\right\}. \end{array}$$

Fig 3K illustrates the migratory region (low miR-451, high AMPK, low mTOR) and proliferative zone (high miR-451, low AMPK, high mTOR) in a *M*/*A*/*R* cube. Fig 3G–3I show the time flow of the dynamics for those three cases (*G* = 0.1, 0.45, 1.0) with two initial conditions: *M*(0) = 2.2, *A*(0) = 3.0, *R*(0) = 2.2 (solid lines; red star (*) in Fig 3A–3F); *M*(0) = 1.8, *A*(0) = 3.0, *R*(0) = 1.8 (dotted lines; green star (*) in Fig 3A–3F)). Glucose withdrawal (*G* = 0.1) induces only one SS (**S**_*low*_ = (0.25, 3.96, 1.44); circle in Fig 3A and 3D). This down-regulation of miR-451 and mTOR, and increased AMPK activity increase the migratory status of the cell. In contrast, the up-regulation of miR-451, and low AMPK activity, and increased mTOR level, leading to a proliferative phase, are induced under normal (high) glucose conditions (*G* = 1.0; Fig 3C and 3F; one SS **S**_*high*_ = (4.11, 0.42, 4.59)). For an intermediate level of glucose (*G* = 0.45; Fig 3B and 3E), the system generates three SS: one unstable SS $S_{mid}^{2}=\left(2.06,0.96,3.28\right)$; dotted circles) in the middle and two stable SS (two solid circles; one $S_{mid}^{3}=\left(2.95,0.61,4.11\right)$ in the proliferative zone (blue solid box) and another one $S_{mid}^{1}=\left(0.76,2.74,1.67\right)$; in the migratory zone (red dotted box)). This leads to a bi-stable system similar to results in the smaller miR-451-AMPK system developed by Kim *et al.* [59, 63].

When the core miR-451-AMPK-mTOR system (1)–(3) is in equilibrium, we can solve miR-451 levels (*M*^*s*^) as a function of the extracellular glucose level (*G*). In a similar fashion, we can also obtain the bifurcation curve of steady state values of AMPK activity (*A*^*s*^), and mTOR levels (*R*^*s*^) w.r.t. external glucose signal (*G*). Fig 3J shows the graphs *M* = *M*(*G*) (blue), *A* = *A*(*G*) (red), *R* = *R*(*G*) (green) as a *S*-shaped curve (hysteresis) with reversed direction of the *A*-curve. While the upper and lower branches of those curves are stable, the middle branch is unstable. Under glucose withdrawal conditions, the system (1)–(3) travels along the lower branch (*M* low, *A* high, *R* low) of the miR-451 curve and the cells are in the migratory phase. The cell continues to migrate as *G* is increased until it reaches the right knee point of the bifurcation curve (∼0.6). Around this point, both miR-451 and mTOR levels jump to the upper branch, with elevated levels of miR-451 and mTOR and down-regulated AMPK, and the cells are put in the proliferative phase (migration switch is turned off).

The size of the bi-stability window ($W_{b}=\left[b_{m}^{w},b_{M}^{w}\right]$) depends on other parameters and may even disappear under the perturbation of some parameters (see S1 Appendix). As *G* is decreased (for example when glucose consumption is increased due to fast tumor growth), the miR-451 and mTOR levels remains elevated and AMPK activity is suppressed, until it reaches the left knee point of the curve (∼0.4), at which time the miR-451 and mTOR levels jump down to the lower branch and AMPK is up-regulated. Then the cell switches to the migratory mode. Therefore, the effect of glucose is history dependent: For an intermediate level of glucose ($0.4=b_{m}^{w}<G<b_{M}^{w}=0.6$; bi-stable mode in Fig 3B), the cells are in the proliferative phase if *G* was in decreasing mode, and in the migratory phase if *G* was in increasing mode. The bifurcation diagram in Fig 3J suggests that a state (*G*, *M*, *R*) with *M* > *th*_*M*_ = 2.0, *R* > *th*_*R*_ = 3.0 will be moved by the dynamic system (1)–(3) into the upper stable branch, resulting in over-expression of miR-451 and mTOR and, thus, cells will be in the proliferation phase. On the other hand, if *M* < *th*_*M*_ = 2.0, *R* < *th*_*R*_ = 3.0, then (*G*, *M*, *R*) will stay in the lower stable state branch, leading to cell migration. This is consistent with results shown in [59] for a smaller miR-451-AMPK system. Fig 3K summarizes the relative locations of the proliferative and migratory phases in state of those key variables (miR-451, AMPK, and mTOR). As mentioned above, the behaviors of mTOR are same as those of miR-451.

See S1 Appendix for more detailed analysis on the miR-451-AMPK-mTOR core control system: (i) Characterization of migration and proliferation under perturbation of key parameters. (ii) Sensitivity Analysis (iii) Therapeutic approaches by using the regulation of migration in glioblastoma via time delays and stochastic effect.

### Regulation of the normal cell cycle and quiescent phase

**Fig 4:**
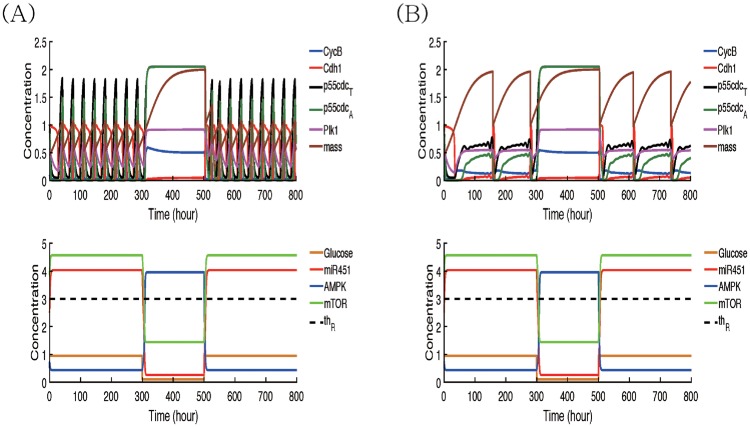
Effect of hypoxia on the dynamics of intracellular signaling pathways. Time courses of cell cycle variables (CycB, Cdh1, p55cdc_*T*_, p55cdc_*A*_, plk1, mass, and mass^*s*^) and miR-451-AMPK-mTOR module in response to high and low glucose levels in normoxia (A) and reduced oxygen (B) conditions. Under a normoxia condition, the cell enters the quiescent phase (G0-phase; [*G*_0_] = 1) when glucose is deprived around *t* = 300 *h*. However, the cell returns to the normal active cell cycle when the glucose level is increased to the high level around *t* = 500 *h*. Under a hypoxic condition, the system leads to a slower cell cycle.

Fig 4 shows time courses of cell cycle variables (CycB, Cdh1, p55cdc_*T*_, p55cdc_*A*_, plk1, mass, and mass^*s*^) and MAR module (miR-451, AMPK complex, and mTOR) in response to fluctuating glucose levels in normoxia (Fig 4A) and hypoxia (Fig 4B) conditions. The cell dynamically changes its cell cycle status in response to the different glucose levels, alternating between high and low conditions. Under a normoxia condition, the initially high glucose level induces upregulation of miR-451 and mTOR levels and downregulation of the AMPK complex, sending a signal for a normal cell cycles (No of cell cycles = 8). When glucose is deprived around *t* = 300 *h*, the cell enters a quiescent phase (G0 phase; [*G*_0_] = 1) via downregulation of miR-451/mTOR levels and upregulation of the AMPK kinases, However, the cell returns to the normal active cell cycle when the glucose level is increased to the normal level around *t* = 500 *h*. Under a hypoxic condition, high glucose levels also induce the slower cell cycle (No of cell cycles = 3) via the miR-451-AMPK-mTOR core control system. Like the normoxia case, glucose deprivation induces the low mTOR (low miR-451, high AMPK) level, leading to G0 phase, but the subsequent escalation of glucose brings the cellular status back to a normal cell cycle through the miR-451-AMPK-mTOR system, escaping the G0 phase.

**Fig 5:**
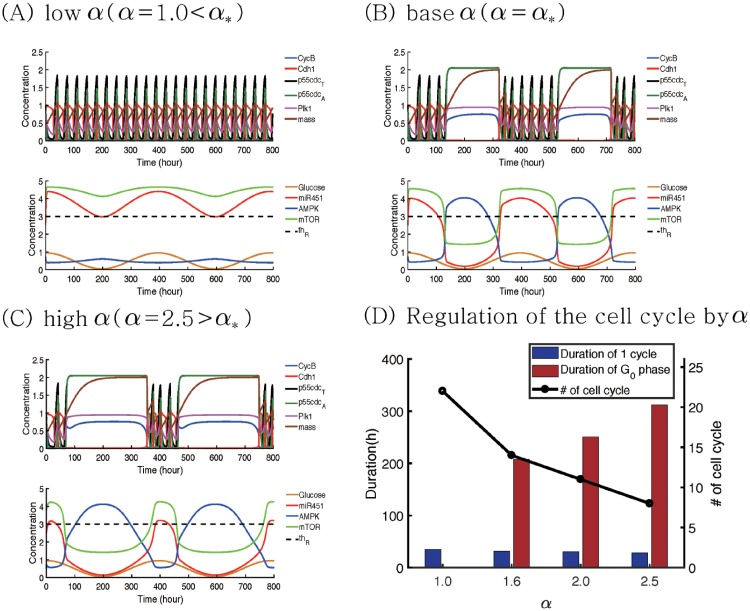
Effect of the inhibition strength *α* on the cell cycle regulation in response to glucose fluctuation. (A-C) Time courses of variables in cell cycle (CycB, Cdh1, p55cdc_*T*_, p55cdc_*A*_, plk1, volume, and mass^*s*^) and miR-451-AMPK-mTOR module in response to a periodically fluctuating glucose level (*G*(*t*) = 0.45*cos(π*t/20) + 0.5) when *α* = 1.0 (low), 1.6 (base), 2.5 (high). When *α* is decreased (*α* = α_*_ → 1.0 in (A)) or increased (*α* = α_*_ → 1.0 in (C)) from the base value (α_*_ = 1.6), the system leads to quantitatively different durations of normal cell cycle and G0-phase. (D) Distribution of normal cell cycles and quiescent phase for various *α*’s (*α* = 1.0, 1.6, 2.0, 2.5). As *α* is increased, average duration of G0-phase (red bar) is increased and the total number of normal cell cycles (black circle) is decreased.

In Fig 5 we investigate the effect of inhibition strength *α* on the normal cell cycle and G0 phase in response to a fluctuating glucose level (0.45*cos(π*t/20) + 0.5). Fig 5A–5C show time courses of variables in cell cycle (CycB, Cdh1, p55cdc_*T*_, p55cdc_*A*_, plk1, mass, and mass^*s*^) and miR-451-AMPK-mTOR module when *α* = 1.0 (low), 1.6 (base; α_*_), 2.5 (high). In the base case, the cell cycle system responds naturally to fluctuating glucose conditions, i.e. transitions between normal cell cycle and G0 phases in response to escalating or descending glucose levels (Fig 5B). For a low value of *α* (*α* = 1.0), the probability of having a proliferation phase is increased and the migration phase is impossible since the bifurcation curves (steady states of miR-451, AMPK complex, and mTOR (*M*^*s*^, *A*^*s*^, *R*^*s*^) as a function of *G*) moves to the left (S1 Appendix). Therefore, the initial glucose level still leads to normal cell cycle via upregulated levels of miR451/mTOR and the corresponding low AMPK activities in the core system but the proliferation phase $T_{p}$ with a normal cell cycle still persists in response to the low glucose level around the time intervals ([180 *h*, 220 *h*]∪[580 *h*, 620 *h*]) due to the sustained levels of miR-451, AMPK complex, and mTOR (*M*(*t*) > 3.0 > *th*_*M*_; *R*(*t*) > 4.1 > *th*_*R*_;*A*(*t*) < 1.0 < *th*_*A*_) as shown in Fig 5A. Therefore, lowering *α* leads to a normal cell cycle regardless of high and low glucose conditions. When *α* is increased (*α* = 2.5; Fig 5C), the system induces the $T_{m}$-phase even for relatively high glucose levels, decreasing the chance of generating a normal cell cycle. Therefore, the enhanced inhibitory activities of miR-451 (*α*) increase the duration of the G0-phase and decrease the duration of the normal cell cycle. This effect of *α* on the G0-phase and normal cell cycle is similar to the impact of *γ* due to the structure of the network and its dynamics (S1 Appendix). Fig 5D shows the distribution of duration of normal cell cycles and G0-phase for various *α*’s (*α* = 1.0, 1.6, 2.0, 2.5). As *α* is increased, the average duration of G0-phase (red bar) is increased and the tumor cell spends more time in the quiescent phase. While the cell cycle period stays almost the same (∼30*h*), the total number of normal cell cycles (black circle) is significantly decreased due to the increased duration of the G0-phase.

**Fig 6:**
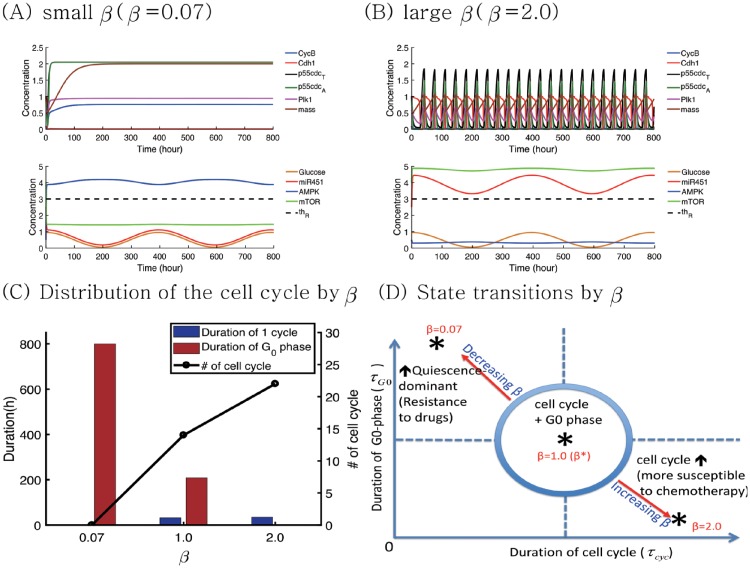
Effect of the inhibition strength *β* on the cell cycle regulation in response to glucose fluctuation. (A,B) Time courses of variables in cell cycle (CycB, Cdh1, p55cdc_*T*_, p55cdc_*A*_, plk1, volume, and mass^*s*^) and miR-451-AMPK-mTOR module in response to a periodically fluctuating glucose level (0.45*cos(π*t/20) + 0.5) when *β* = 0.07 (low), 2.0 (high). When *β* is decreased (*β* = β_*_ → 0.07 in (A)) or increased (*β* = β_*_ → 2.0 in (C)) from the base value (β_*_ = 1.0), the system leads to G0-dominant and full cell cycle system, respectively. (C) Distribution of normal cell cycles and quiescent phase for various *β*’s (*β* = 0.07, 1.0, 2.0). As *β* is increased, average duration of G0-phase (red bar) is decreased and the total number of normal cell cycles (black circle) is increased. (D) Characterization of normal cell cycle and quiescent mode in the *τ*_*cyc*_ − *τ*_*G*0_ plane in response to increase or decrease in *β*.

In Fig 6, we investigate the effect of inhibition strength *β* on the normal cell cycle and G0-phase under the same glucose fluctuation condition (0.45*cos(π*t/20) + 0.5). Fig 6A and 6B show time courses of variables in cell cycle (CycB, Cdh1, p55cdc_*T*_, p55cdc_*A*_, plk1, mass, and mass^*s*^) and miR-451-AMPK-mTOR module when *β* is decreased (*β* = 1.0(β_*_) → 0.07) or increased (*β* = 1.0(β_*_) → 2.0). For a low value of *β* (*β* = 0.07), the bifurcation curve is shifted to the right and the system induces the $T_{m}$-phase in response to high and low glucose levels (S1 Appendix) due to continuous suppressed levels of miR-451 and mTOR, and upregulation of the AMPK complex level (*M*(*t*) < 1.3 < *th*_*M*_; *R*(*t*) < 1.8 < *th*_*R*_;*A*(*t*) > 3.0 > *th*_*A*_)). This induces the continuous duration of the G0-phase in the presence of glucose fluctuation. On the contrary, when *β* is increased to 2.5, the probability of $T_{p}$ (or $T_{m}$) is increased (or decreased). The initial normal cell cycle from the high glucose level persists despite the low glucose level around the time intervals ([180 *h*, 220 *h*]∪[580 *h*, 620 *h*]) because it stays in the upper branch of the bifurcation curve (S1 Appendix). Therefore, the system adapts to full normal cell cycle without entering the G0-phase even in tumor microenvironment with fluctuating glucose supply. Fig 6C shows the distribution of duration of normal cell cycle and G0-phase for various *β*’s (*β* = 0.07, 1.0, 2.0). As *β* is increased, the cell reduces G0-phase (red bar), increasing the total number of normal cell cycles (black circle). Fig 6D illustrates the distinct characteristic changes of the cell cycle mode in the *τ*_*cyc*_ − *τ*_*G*0_ plane when *β* is either increased or decreased. Here, *τ*_*cyc*_, *τ*_*G*0_ are duration of the normal cell cycle and G0 phases, respectively. The enhanced inhibitory activities of the AMPK complex (*β*) may enhance the sensitivity of tumor cells to chemotherapy while an decrease in *β* results in the quiescence-state system.

**Fig 7:**
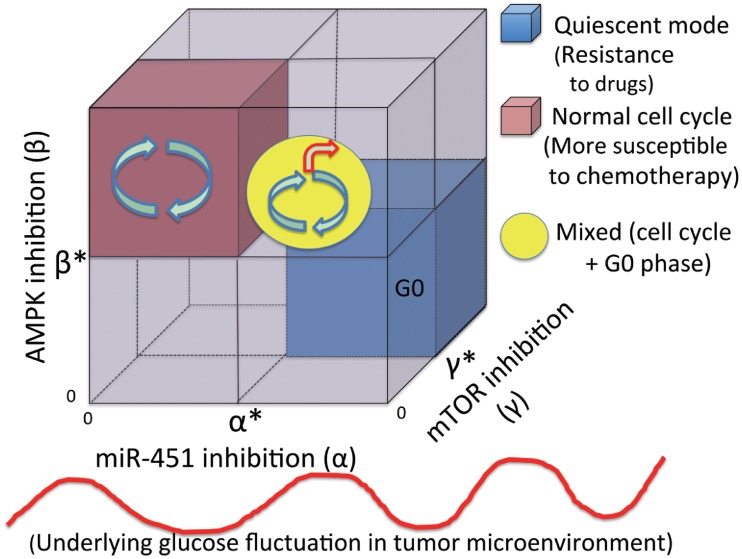
Characterization of cell cycles and quiescent phases. Characterization of normal cell cycle and quiescent modes under the perturbation of *α*, *β*, *γ* in tumor microenvironment where glucose levels fluctuate.

These results suggest that (i) an increase in either *α* (and *γ*) or a decrease in *β* leads to a quiescent mode, which increase resistance to chemotherapeutic drugs. (ii) a decrease in either *α* (and *γ*) or a increase in *β* results in normal cell cycle, which makes the tumor cells more susceptible to a chemo-drug that targets active proliferation. These are summarized in Fig 7. An optimal control theory [104, 105] may be used in optimizing the dose conditions in order to maximize the anti-tumor efficacy by either upregulating or downregulating these signaling molecules while avoiding aggressive invasion and drug complication [106, 107].

### Dynamics of LAR-CSGAG dynamics

**Fig 8:**
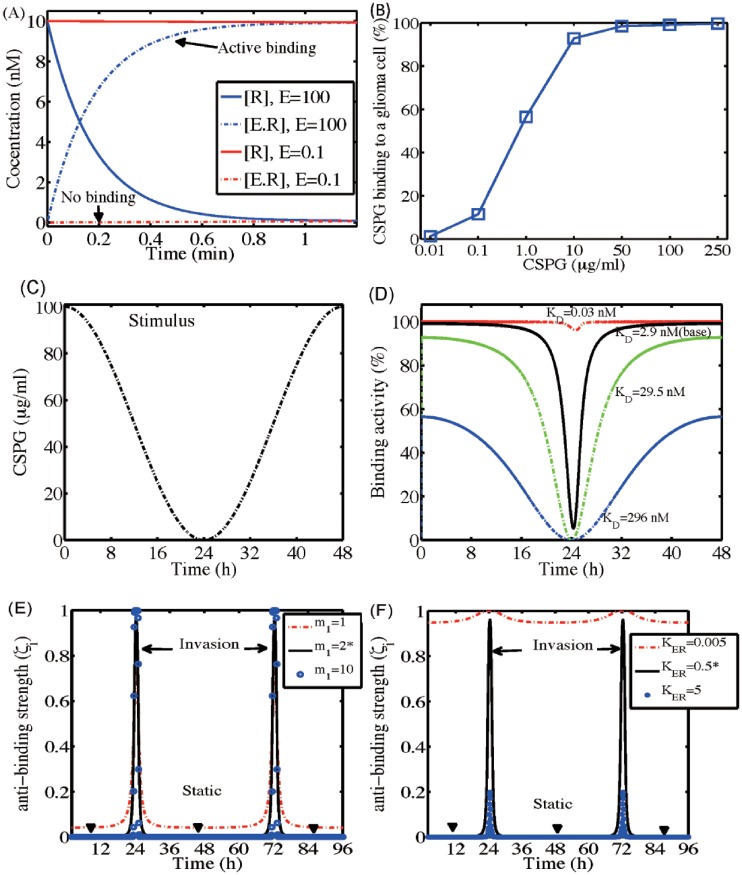
Dynamics of the [CS-GAG]-[LAR] receptor binding. (A) Time courses of concentrations of LAR ([R], solid) and CS-GAGA-LAR complex ([E.R], dotted) in response to high (*E* = 100 *μg*/*ml*) and low (*E* = 0.1 *μg*/*ml*) CSPG stimuli. (B) CSPG binding response. (C,D) Time courses of CSGAG-LAR binding activity (D) for various binding ratios $K_{D}=\frac{k_{off}}{k_{on}}=0.03,2.9,29.5,296\;nM$ in response to fluctuating CSPG input values in (C): (*E*(*t*) = 50*cos(*πt*/24) + 50) in the range of 0-100 *μg*/*ml*. (E,F) Time courses of unbinding strength *ζ*_*i*_ for various Hill coefficients, *m*_1_ = 1, 2(base), 10 in (E) and *K*_*ER*_ = 0.005, 0.5, 5 in (F), in response to the fluctuating CSPG levels *E*(*t*) = 5*cos(*πt*/24) + 5. Initial condition: $R\left(0\right)=10,\bar{E\cdot R}\left(0\right)=0$. Other parameters as in Table 3.

Fig 8A shows time courses of concentrations of LAR ([*R*], solid) and CS-GAGA-LAR complex ([*E*.*R*], dotted) in response to high (*E* = 100 *μg*/*ml*; blue) and low (*E* = 0.1 *μg*/*ml*; red) CSPG levels. While a high CSPG condition induces active and strong binding of a tumor cell to the ECM component by forming the [LAR]-[CSGAG] complex, low levels of CSPG in microenvironment do not warrant this adhesion. Fig 8B shows CSPG binding levels for various CSPG levels (E* = 0.01, 0.1, 1.0, 10, 50, 100, 250 *μg*/*ml*). In general, an increase in CSPG levels induces an increase in the binding. This transient activation of LAR-CSGAG binding is in good agreement with experiments by Fisher *et al.* [78]. In Fig 8C and 8D, we investigate CSGAG-LAR binding activities for various binding ratios $K_{D}=\frac{k_{off}}{k_{on}}=0.03,2.9\left(base\right),29.5,296\;nM$ in response to dynamical changes in CSPG levels (*E*(*t*) = 50*cos(*πt*/24) + 50); Fig 8C). For the base parameter value (*K*_*D*_ = 2.9 *nM*), the strong initial binding activities are weakened as the high CSPG level decreases and this binding force is enhanced again when the CSPG level is increased again. When *K*_*D*_ is small (*K*_*D*_ = 0.03 *nM*; red dashed), the system maintains the high level of the adhesion between a tumor cell and ECM in spite of the fluctuating CSPG levels. When *K*_*D*_ is large (*K*_*D*_ = 296 *nM*), the binding activity is reduced to 60% in response to the high CSPG level and still fluctuates to the changing CSPG stimuli. Fig 8E shows time courses of unbinding strength *ζ*_*i*_ for various Hill coefficients (*m*_1_ = 1, 2(base), 10) in response to the fluctuating CSPG levels *E*(*t*) = 5 * cos(*πt*/24) + 5. Initial condition was $R\left(0\right)=10,\bar{E\cdot R}\left(0\right)=0.$ The usual low unbinding strength from high CSPG levels transits to a peak high value whenever the CSPG level is low, leading to the aggressive invasion of a tumor cell (*m*_1_ = 2* (base); black solid). This suppressed *ζ*_*i*_ (*ζ*_*i*_ = 0, black arrowheads), *i.e.,* strong LAR-CSGAG binding, prevents a tumor cell from invading the surrounding brain tissue in a CSPG-rich microenvironment. For a higher Hill coefficient (*m*_1_ = 10; blue dotted), the invasion is governed by the same CSPG-mediated regulation. For a lower Hill coefficient (*m*_1_ = 1, red dashed), *ζ*_*i*_ shows the similar transient behaviors but the base *ζ*_*i*_ value is increased, leading to the possibility of tumor cell invasion even in the CSPG-rich environment. Fig 8F shows time courses of unbinding strength *ζ*_*i*_ for various *K*_*ER*_ = 0.005, 0.5(base), 5 in response to the fluctuating CSPG levels *E*(*t*) = 5*cos(*πt*/24) + 5. When *K*_*ER*_ is too large (*K*_*ER*_ = 5, blue dotted), the system does not generate enough stimulus of tumor cell invasion in a CSPG-depleted environment around *t* = 24 *h* and *t* = 72 *h*. On the other hand, when *K*_*ER*_ is too small (*K*_*ER*_ = 0.005, red dashed), the system maintains the high level of unbinding strength *ζ*_*i*_, leading to unrealistically persistent invasion stimuli in a CSPG-rich microenvironment.

### CSPGs inhibit tumor invasion and regulate tumor microenvironment in brain

**Fig 9:**
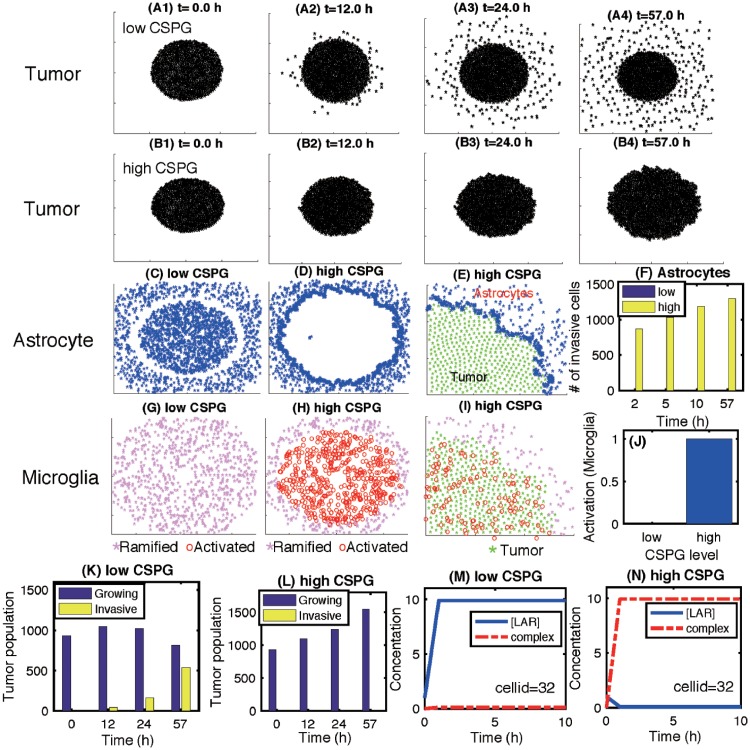
Dynamics of the invasion pattern of glioma cells in response to high and low levels of CSPGs. (A-B) Patterns of growth-invasion of a tumor in the presence of low (2.5 × 10^−2^ *μg*/*mL*) and high (250 *μg*/*mL*) CSPG at time *t* = 0 *h*(A1,B1), 12 *h*(A2,B2), 24 *h*(A3,B3), 57 *h*(A4,B4). (C,D) Patterns of astrocytes at final time (*t* = 57 *h*) in response to low and high CSPGs. (E) A high density of astrocytes (blue asterisk (*)) on the periphery of the tumor in the presence of high CSPG. (F) The number of crossing (invasive) astrocytes in response to low (blue) and high (yellow) CSPGs at time *t* = 2, 5, 10, 57 *h*. (G,H,I) Patterns of ramified (pink asterisk) and activated (blue empty circle) microglia at final time (*t* = 57 *h*) in response to low and high CSPGs. (J) Activation level of microglia in the presence of low and high CSPGs. (K,L) Populations of growing (blue) and invasive tumor cells in response to low and high CSPGs at time *t* = 0, 12, 24, 57 *h*. (M,N) Time courses of concentrations of [LAR] (blue solid) and its complex ([LAR]-[CS-GAG]; red dashed) at a tumor cell site (cellid = 32) in response to low and high CSPGs. Domain size = [60, 140]^2^ in (A,B,C,D,G,H).

Fig 9A and 9B show tumor growth and associated invasion patterns in response to low and high CSPG levels in the computational domain Ω = [0, 1]^2^ at *t* = 0, 12, 24, 57 *h*. While tumor cells actively migrate away from the main tumor core in response to a low CSPG level (Fig 9A), no invasion activities are observed in a CSPG-rich environment (Fig 9B). A low CSPG microenvironment induces the down-regulation of the LAR-CSGAG complex (Fig 9M) and the weak binding between a tumor cell and surrounding ECM, allowing active migration of the tumor cell on the surface of the growing tumor core (Fig 9A). On the other hand, a tumor cell can form a tight adhesion to the neighboring CSGAG ECM in a CSPG-rich environment by up-regulating the LAR-CSGAG complex (Fig 9N), preventing the dispersal of glioma cells into the surrounding brain tissue (Fig 9B). Astrocytes in tumor microenvironment do not physically respond to the low CSPG level (Fig 9C). On the other hand, the heavy CSPG chains also induce active collective movement of astrocytes toward the periphery of the tumor (Fig 9D). Fig 9E shows the subsequent accumulation of astrocytes on the periphery of the growing tumor at the final time (*t* = 57 *h*) in a close-up profile. This dense band of interwoven astrocytes on the boundary of the solid tumor may constrain invasive tumor cells by providing a physical barrier [42]. Fig 9F shows the number of invasive astrocytes at time *t* = 2, 5, 10, 57 *h* in the low (blue) and high (yellow) CSPG conditions. Fig 9G and 9H show the spatial profile of ramified (pink asterisk) and activated (red circle) microglia at final time (*t* = 57 *h*) in response to low and high CSPG levels, respectively. Unlike astrocytes, no physical displacement of microglia is observed in both invasive and noninvasive tumors but distinct and uniform microglial activation states are apparent in spatial domain. While most of microglia are activated within the tumor core, no activation changes of initial ramified microglia are not observed in a low CSPG condition. The high level of CSPG divides the tumor microenvironment into two regions, tumor core region with activated microglia and stromal region with ramified microglia. This critical separation indicates that CSPG plays a central role in activation of the immune cells, microglia (Fig 9J). These different activation status of microglia were observed in glioma [49, 52, 108, 109] as well as other CNS diseases such as Alzheimer’s [110, 111] and Parkinson’s disease [112]. Fig 9K and 9L show populations of growing and invasive tumor cells at time *t* = 12, 24, 57 *h* in response to low and high CSPG levels, respectively. Fig 9M and 9N show time courses of concentrations of [LAR] (blue solid) and its complex ([LAR]-[CS-GAG]; red dashed) at a tumor cell site (cellid = 32) in response to low and high CSPGs. This illustrates that the upregulated LAR-CSGAG complex in response to the high CSPG level is responsible for tight anchoring of a tumor cell to the heavy chain of CSPG ECM.

**Fig 10:**
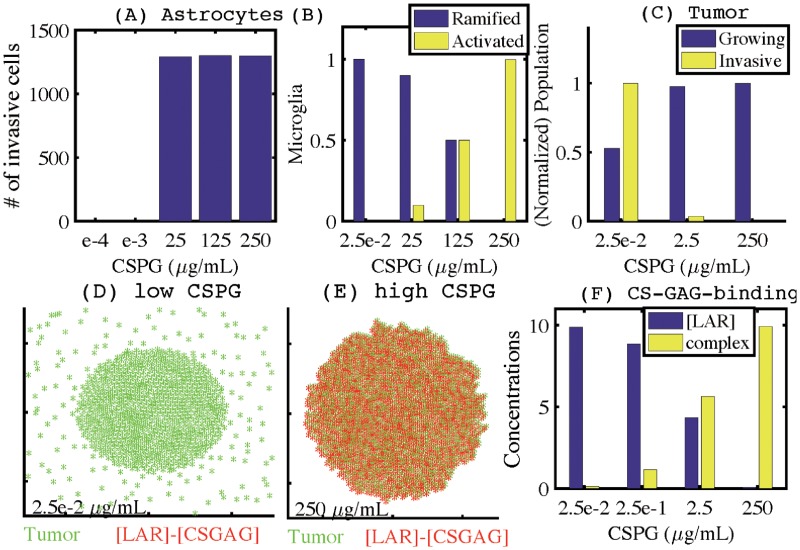
Effect of CSPGs on the spatial dynamics of populations of microglia and astrocytes, and LAR-CSGAG binding. (A) Population of invasive (activated) astrocytes for various CSPG levels (1.0 × 10^−4^, 1.0 × 10^−3^, 25, 125, 250 *μg*/*mL*). (B) Normalized populations of ramified (blue) and activated (yellow) microglia for various CSPG levels (2.5 × 10^−2^, 25, 125, 250 *μg*/*mL*). (C) Normalized population of tumor cells for various CSPG levels (2.5 × 10^−2^, 2.5, 250 *μg*/*mL*). (D,E) Distributions of tumor cells (green star (*)) and [LAR]-[CSGAG] complex (red star (*)) in response to low (2.5 × 10^−2^ *μg*/*mL*) and high (250 *μg*/*mL*) CSPGs. (F) Concentrations of [LAR] (blue) and [LAR]-[CSGAG] (yellow) for various CSPG levels (2.5 × 10^−2^, 2.5 × 10^−1^, 2.5, 250 *μg*/*mL*). Domain size = [70, 130]^2^.

Fig 10A shows the number of reactive (invasive) astrocytes in response to various levels of CSPGs (1.0 × 10^−4^, 1.0 × 10^−3^, 25, 125, 250 *μg*/*ml*). At relatively low concentrations of CSPGs (1.0 × 10^−4^, 1.0 × 10^−3^), the astrocytes remain within the tumor microenvironment. However, with increasing CSPG concentrations (25, 125, 250 *μg*/*ml*), the astrocytes become reactive to the CSPG ECM and move toward the outer tumor boundary. These results suggest that abundant CSPGs are sufficient to induce displacement of astrocytes, leading to a thick wall of astrocytes surrounding the noninvasive tumor. We further tested how tumor-associated CSPGS can change the activation status of microglia within the noninvading lesions. Fig 10B shows the population of ramified (blue) and activated (yellow) microglia in response to various levels of CSPGs (2.5 × 10^−2^, 25, 125, 250 *μg*/*ml*). A low level of CSPGs (2.5 × 10^−2^) induced only ramified microglia. However, as the CSPG concentration increases (25 → 125 → 250 *μg*/*ml*), the population of activated microglia increases and population of the ramified microglia decreases. Those changes in reactive astrocytes (Fig 10A) and activation status of microglia (Fig 10B) as a function of the CSPG level are associated with decreasing population of invasive tumor cells (Fig 10C). Whereas LAR-CSGAG reactions are uniformly observed throughout the CSPG-rich noninvasive tumor (E* = 250 *μg*/*ml*; Fig 10E), it is absent in invasive tumor lesions with a low CSPG concentration (E* = 2.5 × 10^−2^ *μg*/*ml*; Fig 10D). These district uniform distributions of the CSPG receptor molecules (LAR) are in good agreement with experimental observation [42]. The specific LAR-CSGAG binding activities in response to various CSPG levels (2.5 × 10^−2^, 2.5 × 10^−1^, 2.5, 250 *μg*/*ml*) are shown in Fig 10F. In the low CSPG condition, the LAR-CSGAG concentration is down-regulated and the binding activity is suppressed (E* = 2.5 × 10^−2^). However, as the CSPG concentration is increased, the level of LAR-CSGAG complex is increased, leading to a tight adhesion between the glioma cells and their ECM. CSPGs in turn is anchored by this LAR-mediated adhesion within the tumor lesion and resist tumor cell invasion.

**Fig 11:**
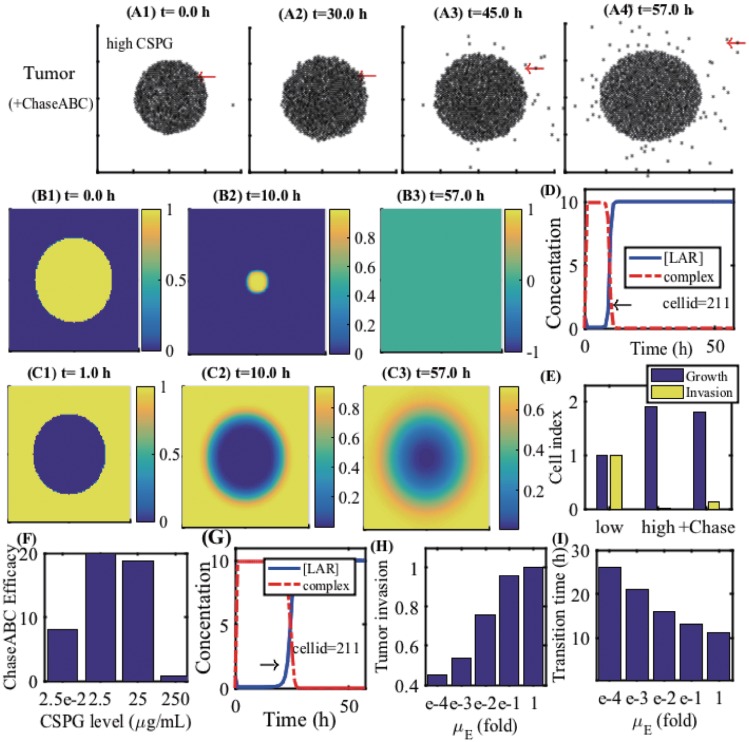
A breakdown of the CS-GAG-mediated tumor-ECM adhesion by Chase-ABC enhances tumor invasion in brain. (A) Spatial profiles of tumor cells at *t* = 0 (A1), 30 (A2), 45 (A3), 57 *h* (A4) in the ChaseABC-treated CSPGs. (B) Spatial distribution of CSPG at *t* = 0 (B1), 10 (B2), 57 *h* (B3). (C) Spatial distribution of Chase-ABC at *t* = 0 (C1), 10 (C2), 57 *h* (C3). (D) Time courses of concentrations of [LAR] and complex ([LAR]-[CS-GAG]) at a tumor cell site (cell id = 211). The location of the cell site was marked in a red arrow in (A). (E) Normalized cell index for growth and invasion of tumor cells at final time (*t* = 57 *h*) in response to low, high, and high + Chase-ABC CSPG levels. (F) Tumor invasion efficacy of Chase-ABC in different CSPG conditions (*E*_0_ = 2.5 × 10^−2^, 2.5, 25, 250 *μg*/*mL*). (G) Time courses of levels of [LAR] and complex ([LAR]-[CS-GAG]) at the same tumor cell site (cell id = 211) under a slower decay condition ($\mu_{E}^{*}=1.0\times 10^{-4}\mu_{E}$). (H) Tumor invasion index for various degradation rates of CSPGs by Chase-ABC: *μ*_*E*_ = 1.0 × 10^−4^, 1.0 × 10^−3^, 1.0 × 10^−2^, 1.0 × 10^−1^, 1-fold. (I) Transition time (*h*) from growth phase to invasion phase at a cell site (cell id = 211) for various degradation rates of CSPGs by Chase-ABC: *μ*_*E*_ = 1.0 × 10^−4^, 1.0 × 10^−3^, 1.0 × 10^−2^, 1.0 × 10^−1^, 1-fold.

We now investigate the effect of a breakdown of CS-GAG-mediated tumor-ECM bonding on tumor invasion. Chase-ABC has been used for degradation of CSPGs in many studies [24, 40, 41, 45, 46]. For example, treatment of the injured spinal cord in the brain with the Chase-ABC cleaves CSPG GAG side chains [113] and alterations in CSPG expression may occur both near and far from the site of spinal injury [114]. Fig 11A shows the spatial profiles of tumor cells at *t* = 0 (A1), 30 (A2), 45 (A3), 57 *h* (A4) when a CSPG-rich ECM is treated by Chase-ABC. Recall that no migratory tumor cells were observed in response to a high CSPG level (Fig 9B). A Chase-ABC-induced breakdown of CSPGs leads to active migration of tumor cells on the surface of the growing tumor core. Fig 11B and 11C show the spatial distribution of CSPG and Chase-ABC, respectively, at *t* = 0 (B1,C1), 10 (B2,C2), 57 *h* (B3,C2). Fig 9D shows time courses of concentrations of receptor LAR and the LAR-CSGAG complex at the site (cell id = 211) of a tumor cell (red arrow in Fig 9A) that was initially located within the tumor core (red arrow in Fig 9(A1). Initially, the LAR-CSGAG complex level is up-regulated in response to a high CSPG concentration (*t* = 0 *h*), preventing the tumor cell from invading the surrounding brain tissue. However, the subsequent Chase-ABC-mediated depletion of CSPGs (Fig 9(B2)) leads to a transition to down-regulation of the LAR-CSGAG complex level (black arrow in Fig 9D) at *t* = 10 *h*, resulting in the release of the tight adhesion to the tumor ECM and aggressive invasion of the tumor cell (Fig 9(A3) and 9(A4)). It is worth noting that this tumor cell (cellid = 211) begins to migrate from the surface of the tumor at a much later time (*t* > 30 *h*) despite the migration signal (the phase transition to down-regulation of the LAR-CSGAG complex) at the earlier time (*t* ∼ 10 *h*). This inhibition of tumor cell migration (cellid = 211) is due to physical constraints from the surrounding tumor cells within the tumor core (Fig 9(A2)). Once the tumor cell is free from other neighboring cells, the tumor cell on the surface of the tumor core initiates the invasion process without the associated mechanical anchoring to the tumor ECM. Fig 9E shows the normalized cell index for growth (blue) and invasion (yellow) of tumor cells at final time (*t* = 57 *h*) in response to low, high, and high+Chase-ABC CSPG levels. Chase-ABC-induced degradation of CSPGs changes the non-invasive tumor type to an invasive tumor. The invasion index in a Chase-ABC-treated tumor is small relative to the low CSPG case due to the delay of CSPG degradation by Chase-ABC. In Fig 9F, we investigate the effect of Chase-ABC treatment on tumor invasion in various CSPG conditions (*E*_0_ = 2.5 × 10^−2^, 2.5, 25, 250 *μg*/*mL*). Chase-ABC may induce a nonlinear dynamics in tumor cell invasion. In general, in the intermediate and low levels of CSPGs (*E*_0_ = 2.5 × 10^−2^, 2.5, 25 *μg*/*mL*), the ratio of Chase-ABC-mediated tumor cell invasion relative to the control case (case without Chase-ABC) is larger than in the high CSPG condition (*E*_0_ = 250 *μg*/*mL*), due to the faster degradation of CSPGs by the Chase-ABC. Fig 9G shows time courses of levels of LAR and the LAR-CSGAG complex at a cell site (cell id = 211) under a slower decay condition ($\mu_{E}^{*}=1.0\times 10^{-4}\mu_{E}$). The phase transition (black arrow) from the strong (up-regulated LAR-CSGAG complex) to weak (down-regulated LAR-CSGAG complex) cell-ECM bond is delayed (*T*_*t*_ = 10*h* → 20*h*), compared to the control case (Fig 9D). In Fig 9H and 9I, we investigate the dynamics of tumor invasion for various degradation rates of CSPGs by Chase-ABC: *μ*_*E*_ = 1.0 × 10^−4^, 1.0 × 10^−3^, 1.0 × 10^−2^, 1.0 × 10^−1^, 1-fold. As *μ*_*E*_ is decreased, the slower degradation of CSPGs delays the transition time (Fig 9I) for weakening of the tumor cell-ECM adhesive bonds, leading to the lower tumor invasion index (Fig 9H).

### Effect of microenvironment on CSPG-mediated tumor invasion

**Fig 12:**
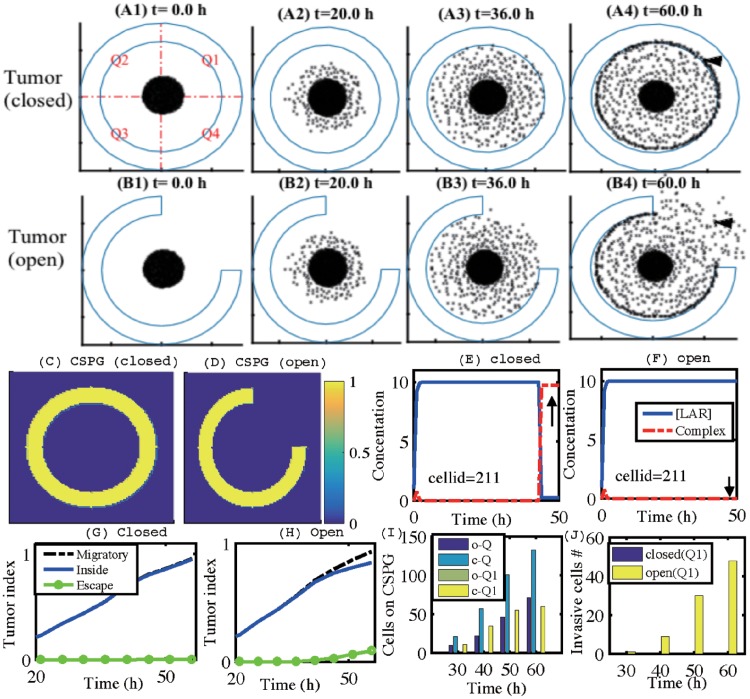
Role of the CSPG microenvironment in regulation of glioma infiltration. (A,B) Time courses of growth/invasion patterns at *t* = 0, 20, 36, 60 *h* in the presence of a thick CSPG ring without (A) and with (B) a partial open section. (C,D) Spatial distribution of CSPG in closed and open cases. (E,F) Time courses of levels of [*LAR*] and [*LAR*] − [*CSGAG*] complex at a cell site (cell id = 211, arrowheads in (A4) and (B4)) in closed and open cases. (G,H) Time courses of tumor index (normalized tumor cell populations): total migratory cells (black dashed), migratory cells inside the ring structure (blue solid), and escaped migratory cells (green circle). (I) Number of tumor cells that adhered to the boundary of CSPG ring region at time *t* = 30, 40, 50, 60 *h*: cells in Q1,Q2,Q3,Q4 regions in the open case (o-Q), cells in Q1,Q2,Q3,Q4 regions in the closed case (c-Q), cells in the Q1 region in the open case (o-Q1), cells in the Q1 region in the closed case (c-Q1). The Q1,Q2,Q3,Q4 regions were defined in (A1). (J) Number of invasive (escaping) tumor cells in the Q1 section at time *t* = 30, 40, 50, 60 *h* in the closed (blue) and open (yellow) cases.

CSPGs, major components of ECM in the brain may form a complex structure such as a patch morphology like a dandelion clock [115] or a ring-like structure with partial opening [114]. Fig 12A and 12B show time courses of growth/invasion patterns at *t* = 0*h* (A1,B1), 20*h* (A2,B2), 36*h* (A3,B3), 60*h* (A4,B4) in the presence of a thick CSPG ring without (Fig 12A) and with (Fig 12B) a partial open section. Four invasion sectors in the domain are marked as Q1 (north-east), Q2 (north-west), Q3 (south-west), Q4 (south-east). See Fig 12A. Fig 12C and 12D show spatial distributions of CSPG levels for circular thick CSPG bands surrounding the tumor at the center of the domain in closed and open cases, respectively. For both cases, tumor cells initially invade the brain tissue in response to low CSPG levels on the periphery of the tumor by down-regulating the LAR-CSGAG complex level. Fig 12E and 12F show time courses of levels of LAR and the LAR-CSGAG complex at a cell site (cell id = 211; arrowheads in Fig 12(A4) and 12(B4)) in closed and open cases, respectively. In the closed case, tumor cell invasion on the front migration wave in all sections (Q1,Q2,Q3,Q4) is inhibited by up-regulation of the LAR-CSGAG complex level (arrow in Fig 12E) on the thick CSPG barrier at a later stage. In the open case, the tumor cells in the Q1 sector invade the brain tissue through the open space (arrowhead in Fig 12(B4)) with sustained downregulation of the LAR-CSGAG complex level (arrow in Fig 12F). Fig 12G and 12H show time courses of normalized tumor cell populations for total migratory cells (black dashed) outside the tumor core, migratory cells inside the ring structure (blue solid), and escaped migratory cells (green circle), in closed and open cases, respectively. Distribution of tumor cell population that adhered to the boundary of the CSPG-rich region at time *t* = 30, 40, 50, 60 *h* are shown in Fig 12I in more detail: cells in Q1,Q2,Q3,Q4 regions in the open (o-Q; dark blue) and closed case (c-Q, light blue), cells in the Q1 region only in the open (o-Q1; green) and closed case (c-Q1; yellow). In the closed case, tumor cells respond to the heavy chain of CSPGs and form strong adhesion to the CSPG ECM uniformly on the boundary of the CSPGs in the all sectors (c-Q) including the Q1 sector (c-Q1). In the open case, less tumor cells are adhered to the CSPG band in the Q2,Q3,Q4 sectors (o-Q) due to a subgroup of invasive tumor cells through the open space in the Q1 sector (o-Q1). Fig 12J shows the number of invasive tumor cells through the Q1 section at time *t* = 30, 40, 50, 60 *h* in the closed (blue) and open (yellow) cases. No migrative tumor cells through the ‘closed’ CSPG-dense loop are observed. These results illustrate the critical role of microenvironmental CSPG distribution in regulation of LAR-dependent glioma invasion. Our results also suggest the possibility of blocking aggressive tumor cell infiltration by rearrangement or injecting of a thick CSPG band on the periphery of a growing tumor, which may suppress the tumor cell infiltration by upregulation of the LAR-CSGAG complex in response to the high CSPG levels.

## Discussion

### GBM

One of the major challenges in treatment of GBM is that by the time the disease is diagnosed glioma cells have already infiltrated into other parts of the brain tissue [4], leading to regrowth of the tumor [9]. Because of its infiltrative nature of growth patterns, guerrilla warriors are often used as a metaphor for diffuse glioma cells [9]. Therefore, better understanding of the fundamental signaling pathways for cell proliferation and migration, and even blocking this critical invasion process, would lead to better clinical outcomes.

### Dynamics of the miR-451-AMPK-mTOR-Cell cycle signaling pathways

miRNAs recently emerged as one of the key regulators of cellular process such as the cell cycle [19, 116]. These miRNAs harbor a clinical significance as therapeutic components in anti-cancer therapy [117–119]. AMPK and mTOR are one of master players in metabolic reprogramming in glioma [120]. Godlewski *et al.* [1, 22] identified a key molecular control system (miR-451, AMPK complex, and mTOR) that provides a critical switch between cell proliferation and migration. We developed a mathematical model of the core control system (miR-451-AMPK-mTOR) based on the experimental observations [1, 22] and analyzed the model’s behavior in response to high and low glucose levels. The responses of the core control system for various glucose levels are in good agreement with experimental results [1, 22]: In particular, (i) the up- and down-regulation of miR-451 and mTOR in response to high and low glucose levels, respectively, and (ii) the down- and up-regulation of the AMPK complex in response to normal and low glucose. This allowed us to define the migratory and proliferative phase based on the status of these molecules and the hysteresis system generates a bistable window (*W*_*b*_ in Fig 3) as predicted in the previous smaller miR-451-AMPK model [59, 63]. The model system predicts oneway-, bistable-, and mono-stability switches under the perturbations of the key inhibition parameters *α*, *β*, *γ* (S1 Appendix). Phenotypic changes under the perturbation of these *α*, *β*, *γ* enabled us to predict the $T_{m}/T_{p}$-status of glioma cells in the glucose fluctuating microenvironment when specific components of the signaling system are perturbed, for example by miR-451-suppressing drugs (S1 Appendix).

We extended our model to take into account cell cycle in glioma cells and showed that the key control of the miR-451-AMPK-mTOR system also determines its downstream of cell cycle, regulating critical switch between normal cell cycle and G0-phase (Fig 4). The mathematical model also predicts that the variations in inhibition of miR-451 (and mTOR) or AMPK activities (*α*, *β*) critically affect the durations of the G0 phase, thus overall cell cycle schedule, in a microenvironment where glucose levels fluctuate (Figs 5 and 6). These also imply that the microenvironment under these changes will either promote or inhibit the susceptibility of tumor cells by cell cycle-targeting drugs as well as duration of the quiescent phase (Fig 7). Many other signaling pathways such as p53, ROS, and autophagy molecules, play a role in regulation of cell cycle and overall metabolism in glioma [121]. For example, autophagy and mitochondrial dynamics influence cell cycle progression [120]. The analysis of the mathematical model in the current paper may serve as a starting point for further experimental investigation and more detailed modeling on extended key networks including PI3K/Akt [122, 123] and VEGF-independent vascularization of GBM [124].

### Role of CSPGs and LAR in regulation of glioma invasion

We investigated the role of CSPG-induced LAR dynamics in regulation of the aggressive invasion of glioma cells using a multi-scale mathematical model. This type of multi-scale hybrid models [60, 61, 84, 85, 125, 126] is useful to describe detailed bio-mechanics of cells (tumor cells, microglia and astrocytes), reaction-diffusion of relevant diffusible molecules, and the corresponding intracellular signaling dynamics. Detailed biomechanical crosstalk between tumor cells and stromal cells (ramified/activated microglia, and astrocytes) through CSPG-induced LAR-CSGAG dynamics. Growth of the tumor cells depends not only the LAR-CSGAG signal status in the core control system but the physical constraints from the neighboring other cells, *i.e.,* inhibition of tumor cell growth and invasion in the interior of the growing tumor mass [61, 84–86]. The model specifically predicts that a CSPG-rich microenvironment is highly associated with a non-invasive tumor (Fig 9B), while the absence or low level of glycosylated CSPGs promotes a diffusely infiltrative type (Fig 9A). Furthermore, the heavy CSPG chains induce upregulation of the LAR-CSGAG complex, providing a strong bond between tumor cells to the ECM (Fig 10E), and astrocyte’s exodus from the dense tumor core (Fig 9D), surrounding the tumor. This strong cell-ECM adhesion and encapsulation of the tumor by accumulated astrocytes prevent tumor cells from invading the surrounding brain tissue. Absence of those two key factors in the CSPG low microenvironment (Fig 9C) triggers the aggressive infiltration of tumor cells by downregulation of the LAR-mediated adhesion (Fig 10D). These results are in good agreement with the experiments [1].

### Role of stromal cells in regulation of glioma invasion

Reactive astrocytes, recognized as a pro-invasive component of the glioma, are reported to promote the proliferation [127] and invasion of brain tumor cells either by secreting tumor-derived connective tissue growth factors [58] and SDF-1 [128], or degradation of a preexisting ECM [129]. While the function of astroglial barriers was reported in the development and injury, the fundamental mechanism of astrocyte-induced inhibition of cellular tumor infiltration remains largely unknown [1]. It was suggested that the encapsulation of the non-invasive tumor by reactive astrocytes could provide a physical barrier to glioma infiltration [1]. Model simulations predict two distinct modes of astrocytes in response to CSPGs: (i) densely packed astrocytes, repelled by the CSPG-rich matrix, may contribute to the non-invasive characteristics of the tumor by inhibiting tumor cell infiltration on the tumor boundary; (ii) in response to a CS-GAG-deplete tumor matrix, more open, sparse field of resident astrocytes allows tumor cell invasion. Reactive astrocytes around spinal cord injury or traumatic brain form a physical barrier to promote inflammation and inhibit the spread of tissue damage in the neighborhood [130–132]. In addition, the molecular agents in injury-associated astrogliotic module are likely present in the astrogliotic wall on the boundary of a non-invasive tumor type [1]. These evidences show the great complexity of the tumor-astrocyte interaction and invasive behaviors of tumor cells. These results also suggest the possibility of using the tumor-confining astrocytes for preventing tumor invasion. When this new strategy is combined with classical therapeutic agents, the overall anti-tumor efficacy may be significantly enhanced. A resection-induced injury after a image-guided surgery was reported to induce spatio-temporal alterations in the population of reactive astrocytes through changes in transcriptome and secretome within the peritumoral lesions, promoting both proliferation and invasion of tumor cells [133]. Therefore, the new anti-invasion strategies with CSPG-astrocytes need to be designed carefully to take into account the changes of these phenotypical changes in astrocytes in response to a conventional surgery.

Microglia may facilitate tumor cell dispersal [42, 49–52, 134] and microglial-derived proteases are highly associated with tumor growth [57]. Our results also indicate the casual association of microglia and tumor invasion. However, the study also indicates the concurrent activation of ramified microglia with the non-invasive tumor dividing a CSPG-rich lesions and outer stroma. The activated phenotype of microglia positioned beyond the periphery of a non-invasive glioma was similar to microglia associated with injury [1], which is reported to suppress the wider spread of damage away from injury sites [135–138]. Therefore, these tumor-associated microglia could be involved in a non-invasive function [1]. The microglial cells has two major subtypes, the inflammatory phenotype M1 and the M2 phenotype and this polarization of M1/M2 depends on many factors in tumor microenvironment in the pathological context [139]. Our results suggest that distinct status of ramified/activated microglia in response to the high and low levels of CSPGs may provide a bi-linear pro- or anti-invasive contributions to the tumor microenvironment (Fig 9G and 9H; Fig 10B). Further experiments and theoretical development are necessary to clarify the exact role of this dual role of microglia in the context of tumor-microglia interaction.

### CSPGs as a therapeutic target

Chase-ABC has been used for changing the tumor ECM condition in various cancer biology such as transition between the invasive and non-invasive phenotypes [42], enhancement of OV spread [40, 41], and its anti-tumor effect [46]. This digestive enzyme was very specific to degradation of CSPGs in the context of the cell-cell adhesion. For example, Silver *et al.* [42] found that the formation of cell aggregation (U-87MG cell line) was not affected by other bacterial enzymes (penicillinase), but consistent monolayer growth was observed only in Chase-ABC-expressing cells. The model predicts that the non-invasive tumor in response to the high CSPG levels transits to invasive phenotype after Chase-ABC treatment by downregulating the level of the LAR-CSGAG complex, exhibiting microscopic tumor cell infiltration on the surface of the tumor mass (Fig 11A). Therefore, the critical CSPG-mediated transition (Fig 11D) between up- and down-regulation of the intracellular binding receptor plays a key role in regulation of invasive and non-invasive tumor phenotypes. The microenvironmental factors such as tissue composition and packing density, and the CSPG concentration also affect the anti-invasion efficacy due to its impact on degradation rate of CSPGs by Chase-ABC (Fig 11(G)–11(I)). Because of the physical constraints, Chase-ABC-mediated tumor cell invasion is limited to the outer surface of the tumor and its impact diminished toward the main tumor core. This suggest the indirect role of Chase-ABC in regulation of the invasive and non-invasive tumors [1].

**Fig 13:**
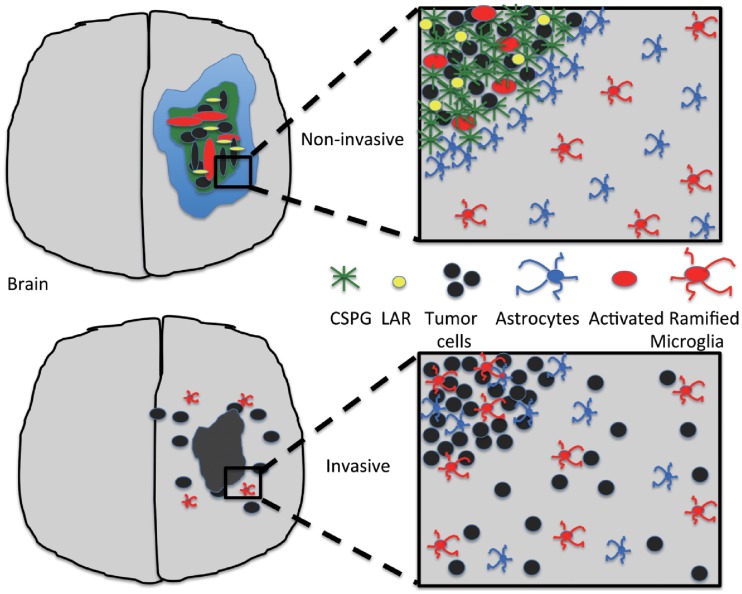
A schematic of CSPG-induced inhibition of tumor invasion via regulation of cell-ECM interaction and stromal cells in brain [42, 162, 163]. A CSPG-rich microenvironment allows tumor cells (black filled circle) to generate the LAR-mediated adhesion to glycosylated CSPG fibrils (green), intrinsically forming a dense non-invasive tumor. Furthermore, abundant CSGAGs repel astrocyte populations (blue) from the tumor core, allowing accumulation of astrocytes on the peritumoral lesion. In tern, these astrocytes may serve as a secondary inhibitor of tumor invasion. Microglia (red circle) are also activated only within the CSPG-occupying dense tumor mass compared to its counterpart, ramified microglia in the invasive areas. Glioma cells are free to invade the surrounding tissue in the absence of both intrinsic (cell-ECM binding) and extrinsic (astrocyte wall) inhibitors of tumor invasion. *CSPG = green asterisk, LAR (CSPG receptor) = yellow circle, tumor cells = black circle, astrocyte = blue, ramified/activated microglia = red.

Glycosylated CSPGs are usually distributed strategically at the particular junction areas of adjacent emerging structures [25–29, 31, 32]. While proteoglycans typically suppress the cell movement away from restricted regions, the fundamental nature of CSPG repulsion is poorly understood. It is a striking observation that CSPGs are uniformly distributed in non-invasive tumors [1] and these non-invasive tumor cells can thrive within the CSPG-rich environment. The overexpression of the CS-GAG receptor LAR in the non-invasive glioma population may explain a mechanism of the strong bond between the glioma cells and the ECM in this inhibitory microenvironment [1]. LARs and their CS-GAG side chains were shown to function as a adhesive agents by tethering tumor cells to proteoglycans [140]. Therefore, the LAR-mediated cell-ECM adhesion may lead to indirect but efficient cell-cell adhesion, and a non-invasive tumor lesion. The reactive astrocytes on the peritumoral microenvironment, repelled from the non-invasive tumor core by CSPGs, may also contribute to blocking tumor invasion as a secondary wall in addition to LAR-CSGAG-induced inhibition. A CSPG-rich microenvironment leads to growing gliomas with compact, non-invasive morphologies (Fig 9). So, surgical resection may be a better option to remove such compact gliomas [141]. Our simulations in the complex distribution of CSPGs show the complex invasive behaviors of tumor cells (Fig 12) in a multi-component tumor microenvironment. Our results also suggest the possibility of CSPG-astrocyte-mediated therapy for blocking aggressive tumor cell infiltration by manipulation or injecting of CSPGs on the periphery of a growing tumor, a strategy that has not yet been explored (Fig 12). Some migratory glioma cells were observed in CSPG-containing microenvironments [142, 143]. However, more and more evidences now suggest that the invasive ECM in glioma samples consists of CSPGs that do not contain CSGAG side chains [42, 144–146]. In this vein, the presence of CSGAG side chain and its receptor LAR, rather than CSPG itself, in tumor microenvironment plays an important role in regulation of non-invasive and invasive glioma (Fig 13). Despite the poor understanding of LAR- and astrocyte-mediated inhibition of tumor invasion, our work shed a novel insight into better understanding of cell-ECM interaction and phenotypical characterization of non-invasive and invasive gliomas in a complex tumor microenvironment.

### Other microenvironmental factors and future work

In this work, we did not take into account many microenvironmental factors such as endogenous immune dynamics [147], signaling networks [148, 149], angiogenesis [148, 150], biophysical interaction between a glioma and blood vessels [148], ECM remodeling for therapy [149, 151–153], or growth factors [154, 155] such as fibroblast growth factors (FGF) [156] and epidermal growth factors (EGF) [49, 157, 158] transforming growth factor-*β* (TGF-*β*) [49, 159], CSF-1 [49, 160], that may play key roles in progress, aggression, invasion of gliomas and development of anti-cancer strategies [161]. However, the multi-scale mathematical model in the current paper is a first step toward further experimental investigation and more detailed modeling by incorporating these microenvironmental factors. We will address these issues in future work.

## Supporting information

S1 Appendix miR-451-AMPK-mTOR system. Development, parameter estimation, analysis, sensitivity analysis, and theoretical implications of the miR-451-AMPK-mTOR core control system.
(PDF)
