# Supplementary material for: Role of extracellular matrix and microenvironment in regulation of tumor growth and LAR-mediated invasion in glioblastoma
Source: PLoS One. 2018 Oct 4;13(10):e0204865. doi: 10.1371/journal.pone.0204865 (PMC6171904; doi:10.1371/journal.pone.0204865)
Supplement: S1 Appendix — Development, parameter estimation, analysis, sensitivity analysis, and theoretical implications of the miR-451-AMPK-mTOR core control system. (PDF) [file pone.0204865.s001.pdf]

S1 Appendix
for "Role of extracellular matrix and microenvironment in regulation of tumor growth and LAR-mediated invasion in glioblastoma"

Yangjin Kim[1,2*], Hyunji Kang[3], Gibin Powathil[4], Hyeongi Kim[3], Dumitru Trucu[5], Wanho Lee[6], Sean Lawler[7], Mark Chaplain[8]
1 Department of Mathematics, Konkuk University, Seoul, Republic of Korea
2 Department of Mathematics, Ohio State University, Columbus, Ohio, United States of America
3 Molecular Imaging Research Center, Korea Institute of Radiological and Medical Sciences, Seoul, Republic of Korea
4 Department of Mathematics, Swansea University, Swansea, United Kingdom
5 Division of Mathematics, University of Dundee, Dundee, United Kingdom
6 National Institute for Mathematical Sciences, Daejeon, Republic of Korea
7 Department of Neurosurgery, Brigham and Women's Hospital, Boston, Massachusetts, United States of America
8 School of Mathematics and Statistics, Mathematical Institute (MI), University of St Andrews, St Andrews, United Kingdom
∗ **E-mail: ahyouhappy@konkuk.ac.kr**

The core control system (miR-451-AMPK-mTOR)

Derivation of the miR-451-AMPK-mTOR model

Using a mathematical model abstracted from the complex network (Fig 1), we illustrate the mechanisms and conditions under which these miR-451 properties operate. A mathematical model based on a system of ordinary differential equations is used. In order to incorporate the signaling network we simplified the network as shown in Fig 1A as follows: We merged all the AMPK regulatory network between CAB39/LKB1/STRAD and AMPK/MARK into one component (AMPK complex; middle box with solid line in Fig 1A) while we kept miR-451 and mTOR (dotted boxes in Fig 1A) in separate modules. The mathematical model network is shown in Fig 1B. We refer to the interactions represented by edges in Fig 1B as the core miR-451-AMPK-mTOR control system. By convention, the kinetic interpretation of arrows and hammerheads in the network represents induction (arrow) and inhibition (hammerhead).

Let the variables m, a and r be activities of miR-451, AMPK complex, and mTOR, respectively. The scheme includes autocatalytic activities of miR-451 (m), AMPK complex (a), and mTOR (r) as reported in [1], microRNA/protein degradation of those key molecules, mutual inhibition between miR-451 and AMPK complex and inhibition of mTOR activity by AMPK complex. Based on biological observations, we write the phenomenological equations for the rate change of those key modules (m, a, r) as follows:

$$\frac{dm}{dt} = f(g) + \frac{\Lambda_1 \Lambda_2^2}{\Lambda_2^2 + \Lambda_5 F(a)} - \mu_1 m, \tag{1}$$

$$\frac{da}{dt} = h_1(s_1) + \frac{\Lambda_3 \Lambda_4^2}{\Lambda_4^2 + \Lambda_6 H_1(m)} - \mu_2 a, \tag{2}$$

$$\frac{dr}{dt} = h_2(s_2) + \frac{\Lambda_7 \Lambda_8^2}{\Lambda_8^2 + \Lambda_9 H_2(a)} - \mu_3 r, \tag{3}$$

where Λ_g is the signaling rate from glucose to miR-451, g is the glucose level, s_1, s_2 are the signalling pathways to AMPK complex and mTOR, respectively, $\Lambda_1, \Lambda_3, \Lambda_7$ are the autocatalytic enhancement parameters for miR-451, AMPK complex, mTOR, respectively and $\Lambda_2, \Lambda_4, \Lambda_8$ are the Hill-type inhibition saturation parameters from the counter part of miR-451, AMPK complex, mTOR, respectively, Λ_5 is the

inhibition strength of miR-451 by the AMPK complex, Λ_6 is the inhibition strength of the AMPK complex by miR-451, Λ_9 is the inhibition strength of the mTOR by the AMPK complex, and finally μ_1, μ_2, μ_3 are microRNA/protein degradation rates of miR-451, AMPK complex, and mTOR, respectively.

As indicated in Eq (1), the signal g increases the rate of miR-451 activation through the function $f(g)$ in the first term, while AMPK-dependent inhibition of miR-451 is expressed through the function $F(a)$ in the denominator of the second term. A simple requirement of these functions is that $\frac{\partial f}{\partial g} > 0$ for all non-negative g, and $\frac{\partial F}{\partial a} > 0$ for all non-negative a. Similarly, the first term $h_1(s_1)$ on the right-hand side of Eq (2) represents an increase in AMPK activity induced by the signal s, and miR-451-dependent suppression of AMPK activity is explored through the function $H_1(m)$ in the denominator. In the same manner, the first term $h_2(s_2)$ on the right-hand side of Eq (3) represents an increase in mTOR activity induced by the signal s_2, and AMPK-dependent suppression of mTOR activity is accounted for through the function $H_2(a)$ in the denominator. One must also have $\frac{\partial h_1}{\partial s_1} > 0$, $\frac{\partial h_2}{\partial s_2} > 0$, $\frac{\partial H_1}{\partial m} > 0$, $\frac{\partial H_2}{\partial a} > 0$ for all non-negative s_1, s_2, m, a. Based on biological observations (Fig 1A), we assume that

$$f(g) = \Lambda_g g,\ F(a) = a^2,\ h_1(s_1) = s_1,\ H_1(m) = m^2,\ h_2(s_2) = s_2,\ H_2(a) = a^2. \tag{4}$$

Furthermore, performing now the following non-dimensionalization

$$\begin{aligned} &T = \mu_1 t,\ M = \frac{m}{m^*},\ A = \frac{a}{a^*},\ G = \frac{g}{m^*},\ \lambda_g = \frac{\Lambda_g}{\mu_1},\ S_1 = \frac{s_1}{\mu_2 a^*},\ S_2 = \frac{s_2}{\mu_3 r^*},\ \lambda_1 = \frac{\Lambda_1}{\mu_1 m^*}, \\ &\lambda_2 = \Lambda_2,\ \lambda_3 = \frac{\Lambda_3}{\mu_2 a^*},\ \lambda_4 = \Lambda_4,\ \lambda_5 = \frac{\Lambda_7}{\mu_3 r^*},\ \lambda_6 = \Lambda_8,\ \alpha = \Lambda_5 (a^*)^2,\ \beta = \Lambda_6 (m^*)^2, \\ &\gamma = \Lambda_9 (a^*)^2,\ \epsilon_1 = \frac{\mu_1}{\mu_2},\ \epsilon_2 = \frac{\mu_1}{\mu_3}, \end{aligned} \tag{5}$$

from the dimensional Eqs (1)-(4) we obtain the associated dimensionless equations with a smaller set of essential control parameters, namely:

$$\frac{dM}{dt} = \lambda_g G + \frac{\lambda_1 \lambda_2^2}{\lambda_2^2 + \alpha A^2} - M, \tag{6}$$

$$\epsilon_1 \frac{dA}{dt} = S_1 + \frac{\lambda_3 \lambda_4^2}{\lambda_4^2 + \beta M^2} - A, \tag{7}$$

$$\epsilon_2 \frac{dR}{dt} = S_2 + \frac{\lambda_5 \lambda_6^2}{\lambda_6^2 + \gamma A^2} - R. \tag{8}$$

Parameter estimation

miRNAs are typically more stable than their targets [2,3] and the typical half-life of a miRNA is much larger than that of AMPK [4,5] or mTOR, leading to the small values of the fractions $\epsilon_1 = \frac{\mu_1}{\mu_2} = 0.02$, $\epsilon_2 = \frac{\mu_1}{\mu_3} = 0.02$ [6–8]. From the estimated concentrations of miRNAs ($80\ pM - 2.2\ \mu M$) in an animal cell (assuming 1,000-25,000 μm^3 volume) [9], we take $m^* = 1.0\ \mu M$. The normal (high; 4.5 g/l) and low (0.3 g/l) glucose levels were used in [1] in order to test the effect of glucose on the miR451 expression and AMPK activities, and proliferation/migration patterns of glioma cells. Using this range of glucose levels ($0.3 - 4.5\ g/l$) in [1] and the miR-451 reference value (m^*), we determined the glucose signaling strength, $(2.4 \times 10^{-5} - 2.4 \times 10^{-3})\ \mu M/h$, resulting in a range of dimensionless glucose input level $\lambda_g G = \frac{\Lambda_g g}{\mu_1 m^*} = 0.01 - 1.0$. Using the reference value of AMPK, $a^* = 100nM$ taken from the measured values (35-150 nM) in rat liver [10], and taking the signal source of the AMPK complex, $s_1 = 2.4\ nM/h$, we estimate the dimensionless value of signaling strength to the AMPK complex to be $S_1 = \frac{s_1}{\mu_2 a^*} = 0.2$ [6,7]. By observing the same patterns of up- and down-regulation of mTOR as those of miR-451 in response to various glucose levels, we take slightly larger values of $S_2 = \frac{s_2}{\mu_3 r^*} = 1.2$ in the work. We

take the dimensionless parameter value $\lambda_1 = \frac{\Lambda_1}{\mu_1 m^*} = 4.0$ by assuming that the autocatalytic rate (Λ_1) of miR-451 is at least 4-fold larger than its negative contribution ($\mu_1 m^*$) from its decay/consumption in the absence of the inhibitory pathway from the AMPK complex [6, 7]. Relying on the observed mutual antagonism between miR-451 and AMPK complex, we also take $\lambda_3 = \frac{\Lambda_3}{\mu_2 a^*} = 4.0$ in a similar fashion. The Hill-type coefficients $\Lambda_2, \Lambda_4, \Lambda_6$ (and their corresponding dimensionless parameters $\lambda_2, \lambda_4, \lambda_6$) are fixed to be unity [6, 7]. Once the parameters above were determined, we fitted the data in the level of LKB1/AMPK activity in [1] in response to negative control and over expressed levels of miR-451 (Fig 5B in [1]) in the following way: The steady state of AMPK complex (A^s) in terms of miR-451 level (M^s) and other parameters in the miR-451-AMPK model can be rewritten by

$$A^s = S_1 + \frac{\lambda_3 \lambda_4^2}{\lambda_4^2 + \beta (M^s)^2} = 0.2 + \frac{4}{1 + \beta (M^s)^2}. \tag{9}$$

The up-regulated AMPK complex level (∼500 *pmol* of phosphate incorporated in a dimensional form) for negative control of miR-451 was down-regulated (∼100 *pmol* of phosphate incorporated in a dimensional form). Using Eq (9) above, we estimated the parameter value of β to be 1.0 which gives the low AMPK complex activity ($\sim A^s = 0.9$) in response to a high dimensionless miR-451 level ($M^s = 4.2$) and the high AMPK complex activity ($A^s = 4.2$) in response to negative control of the miR-451 level ($M^s = 0.0$), resulting in a reasonable ∼4.7-fold difference in the AMPK complex activities as in the experiments in [1]. Finally, by observing the behavior of the system and experimental data, we assume that the inhibition strength ($\alpha = 1.6$) of miR-451 by the AMPK complex is slightly larger than the inhibition strength ($\beta = 1.0$) of the AMPK complex by miR-451 [6,7] while keeping the same value for the inhibition strength ($\gamma = 1.0$) of mTOR by the AMPK complex.

Analysis of the core control system

Characterization of migration and proliferation under perturbation of key parameters

In Fig S1, we show sensitivity of variables in the core control system in response to glucose levels (G) to changes in inhibition strength of miR-451 by AMPK complex (α=1.0, 1.6, 2.0, 2.5). As α is increased, the bifurcation curves of all variables (miR-451, AMPK, mTOR) shift to the right (Figs S1A-S1C). Overall, this increase in α also moves the bi-stability window (W_b) to the right in all cases of miR-451, AMPK, and mTOR and leads to a decrease in the size of the window ($|W_b|$), which is also illustrated in Fig S1D. This indicates that the probability of switching to the migration phase is increased when the inhibition strength of miR-451 by AMPK (α) is increased. For example, the cell is already in the migratory phase ($M < th_M, A > th_A, R < th_R$) when $G = 0.7$ in the case of higher α ($\alpha = 2.5$) while it is still in the proliferative phase ($M > th_M, A < th_A, R > th_R$) in the base case ($\alpha^* = 1.6$). On the other hand, glioma cells would grow even under glucose withdrawal conditions when α is decreased. For example, the cell is still in the proliferative phase ($M > th_M, A < th_A, R > th_R$) for a low glucose level ($G = 0.2$) when α is lowered ($\alpha = 1.0$) while it should be in the migratory phase ($M < th_M, A > th_A, R < th_R$) in the base case ($\alpha^* = 1.6$). Fig S1D shows a phase diagram of $G - \alpha$ for switching behaviors including a monostable region and bistable and one-way switches. An increase in the inhibition strength of miR-451 by AMPK (α) induces a transition from a one-way switch to a bistable switch and to a mono-stability. A decrease in α increases the size of the bi-stability window ($|W_b|$). We recall that, for the base case ($\alpha^* = 1.6$), the existence of bi-stability regimes illustrates that glucose can regulate the forward transition from the migratory phenotype ($\mathbb{T}_m$) to the proliferative phenotype ($\mathbb{T}_p$) and the recurrent process from the $\mathbb{T}_p$-state to the $\mathbb{T}_m$-state. However, when miR-451 activity is highly enhanced with the decreased inhibition signal from AMPK complex (a decrease in α), glucose may regulate only forward transition from $\mathbb{T}_m$-state to $\mathbb{T}_p$-state (one way switch) and a monotonic decrease in glucose does not push cells to the migratory

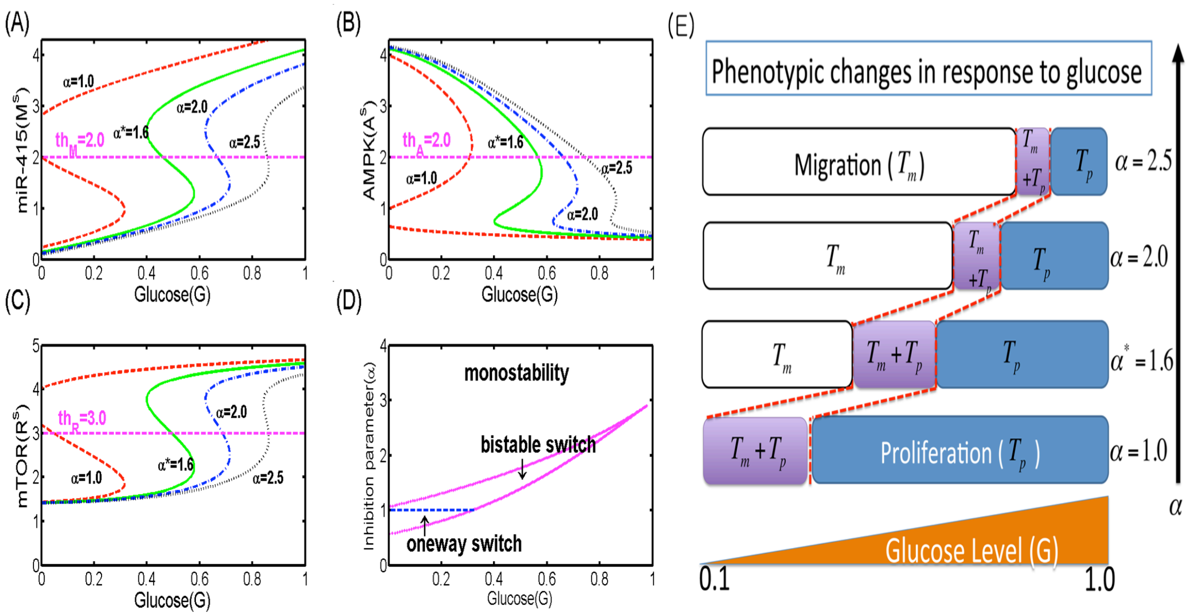

Figure S1. Effect of the miR-451 inhibition strength (α) on the core control system. (A-C) Steady state values of miR-451 expression (M^s in (A)), AMPK activity (A^s in (B)), and mTOR levels (R^s in (C)) in response to glucose levels ($G \in [0, 1]$). (D) Diagram of bistable and oneway switches in G-α plane. Middle dotted line $\alpha = 1.068$ for the boundary between bistable and oneway switches. Other parameters are fixed as in Table 1. The base case ($\alpha^* = 1.6$) was marked in star in (A-C). (E) Phenotypic changes in response to glucose levels (G) when the key inhibition parameters (α) is varied.

phase. Therefore, this mechanism may provide a way of keeping glioma cells in a proliferative phase for a certain period of time in order to prevent the invasion process for second surgery [7]. The phenotypic changes between $\mathbb{T}_m$-state and $\mathbb{T}_p$-state for increased or decreased α is illustrated in Fig S1E. An increase (or decrease) in α generates higher probability of switching to the migratory (or proliferative) phenotype for given glucose level by moving the bifurcation curve and bistable window (W_b) to the right or left.

In Fig S2(A-D) we show the effect of the inhibition strength of AMPK complex by miR-451 ($\beta = 0.07, 1.0, 2.5$) on the dynamics of the core control system. As β is increased, the bifurcation curves of all variables (miR-451, AMPK, mTOR) shift to the left (Figs S2(A-C)). In contrast to the case of changes in α, this increase in β reverses its shifting direction *i.e.,* it moves the bi-stability window (W_b) to the left in all cases of miR-451, AMPK, and mTOR and leads to an increase in the size of the window ($|W_b|$) despite disappearance of the bistable switch, which is also illustrated in Fig S2D. This indicates that the probability of switching to the proliferative phase is increased in response to fluctuating glucose levels ($0 \leq G \leq 1.0$) when the inhibition strength of miR-451 by AMPK (β) is increased. For example, the cell is already in the proliferative phase ($M > th_M, A < th_A, R > th_R$) when $G = 0.2$ in the case of higher β ($\beta = 2.5$) while it should still be in the migratory phase ($M < th_M, A > th_A, R < th_R$) in the base case ($\beta^* = 1.0$). On the other hand, glioma cells would initiate the motility machinery even under normal glucose conditions when β is decreased. For example, the cell would increase probability of switching to the migratory phase by lowering mTOR levels for a normal glucose level ($G = 1.0$) when β is lowered ($\beta = 0.07$) while it should be in the proliferative phase ($M > th_M, A < th_A, R > th_R$) in the base case ($\beta^* = 1.0$). Fig S2D shows a phase diagram of $G - \beta$ for switching behaviors including a monostable region and bistable and one-way switches. An increase in the inhibition strength of AMPK by miR-451

(β) induces a transition from a bistable switch to a one-way switch and to a mono-stability. A decrease in β reduces the size of the bi-stability window ($|W_b|$) but an increase in β reduces the size of the one-way switch window. When miR-451 activity is highly enhanced with the increased inhibition signal of AMPK by miR-451 (an increase in β) due to mutual antagonism between miR-451 and AMPK, glucose may also regulate only forward transition from $\mathbb{T}_m$-state to $\mathbb{T}_p$-state (one way switch) and a monotonic decrease in glucose does not push cells to the migratory phase. Therefore, this mechanism has a similar effect on keeping high miR-451 levels as that of a decrease in α, providing a way of keeping glioma cells in a proliferative phase. In Fig S2E we summarize the phenotypic changes between $\mathbb{T}_m$- and $\mathbb{T}_p$-groups as β is varied. An increase (or decrease) in β leads to weakening (or strengthening) of AMPK module and up-regulation (or down-regulation) of miR-451 due to mutual antagonism between miR-451 (M) and AMPK (A), resulting in cell proliferation (or migration).

Fig S2F illustrates the effect of the increased and decreased inhibition strength of mTOR activity by AMPK complex ($\gamma = 0.08, 1.0, 9.0$) on the regulation of mTOR expression. Recall that cell migration and proliferation heavily depend on glucose levels in the base parameter set, *i.e.,* up- and down-regulation of mTOR expression levels in response to high and low glucose levels (G). However, we found that the dynamics of proliferation and migration are changed when γ is varied. As γ increases from the base value ($\gamma^* = 1.0$), mTOR activity is significantly reduced, leading to underexpression of mTOR regardless of high and low glucose levels. Therefore, enhanced inhibition of mTOR by AMPK complex ($\gamma > \gamma^* = 1.0$ induces complete blocking of mTOR activity ($R < th_R,\ \forall G \in [0, 1]$) followed by cell migration even in the case of high glucose injections ($\gamma = 9.0$). On the other hand, cell migration may be inhibited when γ is decreased from the base value. For example, mTOR levels stay above the threshold value ($th_R = 3.0$) even at low glucose levels ($G = 0.2$ for example) when $\gamma = 0.08$. This means glioma cells may stay in the proliferative phase in response to high and low glucose levels when the inhibition of mTOR by AMPK complex is weakened. We also note that bifurcation curves of two other key molecules (miR-451 and AMPK), thus bi-stability windows, are not affected by the changes in γ due to the network structure of the signaling pathway in the signaling pathway (Fig 1). See Fig S2G. The corresponding phenotypic changes between $\mathbb{T}_m$- and $\mathbb{T}_p$-groups are shown in S2H. As in the case of α, an increase (or decrease) in γ generates higher probability of switching to the migratory (or proliferative) phenotype for a given glucose level by lifting the bifurcation curve down (or up) but keeping the bistable window (W_b).

We recall that the system exhibits a switching behavior, that is, an increase (or decrease) in G induces a transition from $\mathbb{T}_m$-status (or $\mathbb{T}_p$-status) to $\mathbb{T}_p$-status (or $\mathbb{T}_m$-status). Steady-state mTOR bifurcation diagrams as a function of the parameter G for various values of λ_1 are presented in the top panels of Fig S3. It shows the pushing and pulling properties of mTOR (see Figs S3(A-C)). The mTOR level (R^s) decreases with increasing α for the fixed G and λ_1 and the bifurcation curve moves to the right for the fixed λ_1 as seen in Fig S1. While the system exhibits the hysteresis (property of bistable systems) for the base parameter set and the increased λ_1 and various perturbed α's considered ($\lambda_1 = 5.0, \alpha = 1.0, 1.6, 2.0, 2.5$). However, when λ_1 is decreased, for larger values of α, the response of the switch is slightly different. For example, the core control system loses the bi-stability property for larger α's ($\alpha = 1.6, 2.0, 2.5$) when $\lambda_1 = 3.0$ (Figs S3A and S3D). Therefore, even for the base value of α ($\alpha^* = 1.6$) in this case, an increase (or decrease) in glucose leads to a smooth transition from $\mathbb{T}_m$-status (or $\mathbb{T}_p$-status) to $\mathbb{T}_p$-status (or $\mathbb{T}_m$-status) along the branch. However, the system also illustrates an extreme case of hysteresis, a one-way switch, when λ_1 is increased (λ_1=5.0 in Fig S3C) where the upper branches ($\mathbb{T}_p$-regime) stretches into the larger domain but actually need to be eliminated due to physiologically irrelevant regimes ($G < 0$) of glucose (for example, α=1.0 in Fig S3B and α=1.0,1.6 (base) in Fig S3C). In this oneway switching case, glucose withdrawal from the normal level would not induce cell migration from the main tumor mass but the slow supply of glucose from the complete withdrawal condition would put the glioma cell in both $\mathbb{T}_m$- and $\mathbb{T}_p$-status depending on the initial condition of variables (M, A, R) in the core control system. For instance, see the up- or down-regulation of mTOR in response to the slow decrease ($G = 0.0 \to G = 0.5$) or increase ($G = 1.0 \to G = 0.1$) in glucose along the curve ($\alpha = 1.0$) in Fig S3C. Figs S3D-S3F show the

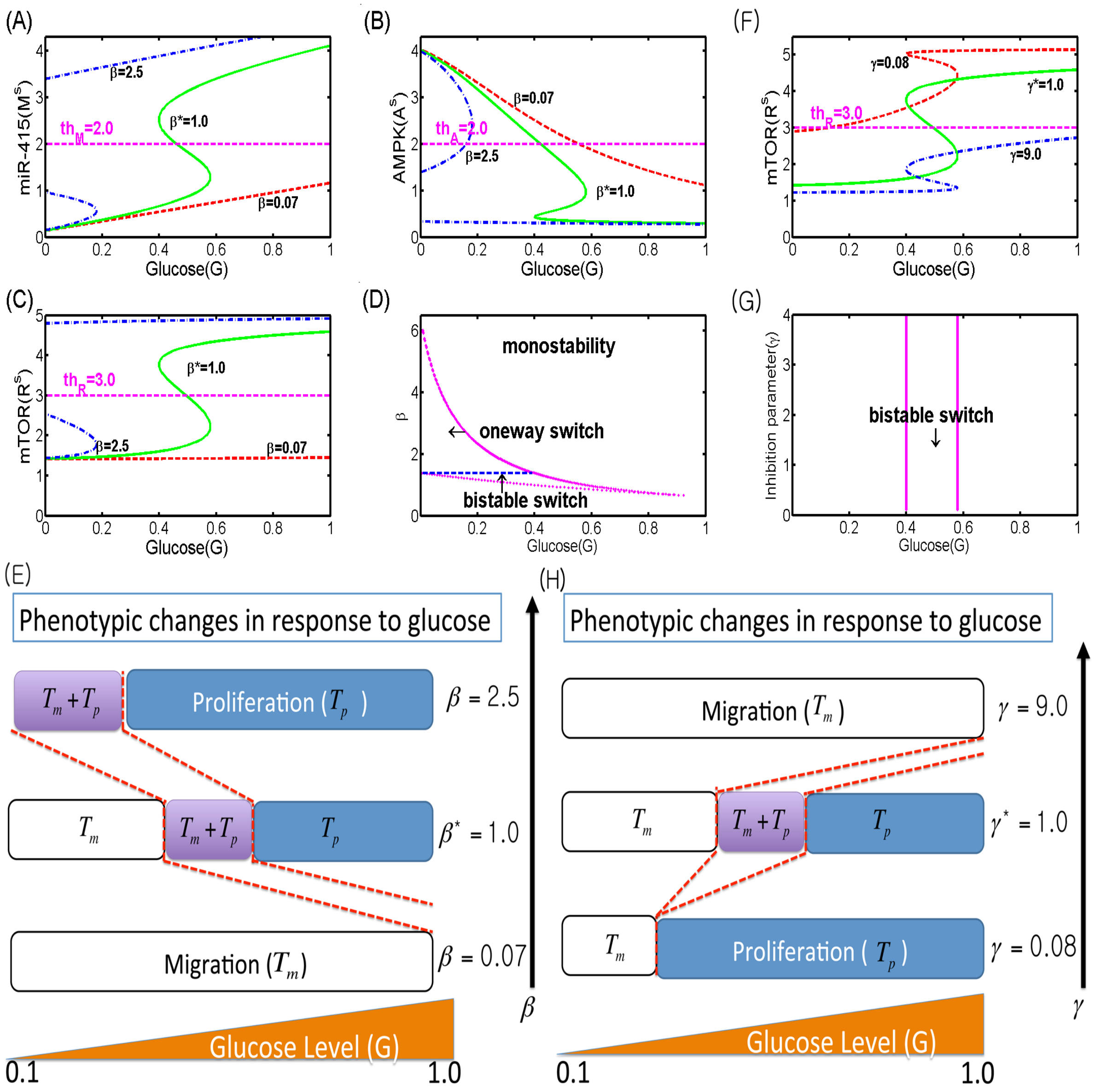

Figure S2. Effect of inhibition strengths β, γ on the dynamics of the core control system. (A-C) Steady state values of miR-451 (M^s in (A)), AMPK complex (A^s in (B)), and mTOR (R^s in (C)) as a function of glucose level (G) for various inhibition strengths of AMPK by miR-451 (β) in the core control system. (D) Diagram of bistable and oneway switches in the G-β plane. The dotted line illustrates the boundary ($\beta = 1.38$) between oneway and bistable switches. Bistability disappears at $\beta = 0.67$. (E) Phenotypic changes in response to glucose levels (G) when β is varied. (F) Steady state values of mTOR (R^s) as a function of glucose level (G) for various inhibition strengths of mTOR activity by AMPK (γ) in the core control system. (G) Diagram of bistable and oneway switches in the G-γ plane. The dotted line shows the boundary ($\beta = 1.38$) between oneway and bistable switches. (H) Phenotypic changes in response to glucose levels (G) when γ is varied. Other parameters are fixed as in Table 1.

corresponding profiles of the bifurcation curves of R^s as a function of glucose levels (G) for different λ_1's (λ_1=3, 4, 5). A bistable area is enclosed by two saddle-node bifurcation points (the right and left knees of the curves in the top panels of Fig S3). For any glucose levels in the bi-stability window $W_b = [b_m^w, b_M^w]$, the system has two stable solutions and one unstable solution as we saw before for the basic parameter set (Fig 3J). The bottom panels of Fig S3 show phase diagrams of switching behaviors including a monostable region and bistable and one-way switches for different λ_1 values. In all three cases, an increase in the inhibition strength of the miR-451 (α) induces a transition from a one-way switch to a bistable switch, and to a mono-stability. An increase in the autocatalytic production rate of miR-451 (λ_1) results in an increase in the size of both oneway and bistable regions in $\alpha - G$ planes. It also moves up the bistable switch region. Therefore, the increased value of λ_1 creates a rich dynamic mixture of oneway switch, bistable switch, mono-stability of the core control system, thus, complex phenotypic switching between $\mathbb{T}_p$- and $\mathbb{T}_m$-types.

In the top panels of Fig S4 we present steady-state mTOR bifurcation diagrams as a function of the glucose level (G) for various values of β (β=0.8,1.0,2.0). The mTOR level (R^s) decreases with increasing λ_3 for the fixed G and β. An increased autocatalytic production rate of AMPK (λ_3: 2.5 $\rightarrow$ 4.0 $\rightarrow$ 5.5 in Figs S4A-S4C) pushes the bifurcation curve to the right for all fixed β's, increasing probability of establishing the $\mathbb{T}_m$-phenotype ($R > th_R = 3.0$). As shown in Fig S2, an increase in β causes an overall shift to the left of those curves, leading to higher chances of acquiring the $\mathbb{T}_p$-phenotype. The core control system loses the bi-stability property, leading to a oneway switch when λ_3 is decreased ($\lambda_3 = 2.5$). Therefore, this has the same effect on cell fate as a decrease in α above (Figs S3B and S3C), *i.e.,* glucose withdrawal does not guarantee the initiation of glioma cell migration from the main tumor core but the glucose supply from the zero level may generate both $\mathbb{T}_m$- and $\mathbb{T}_p$-phenotype depending on initial conditions of those key molecules (miR-451, AMPK, mTOR) in the core control system. The bottom panels of Figs S4D-S4F show the phase diagrams of switching behaviors including a monostable region and bistable and one-way switches for different β's (β=0.8, 1.0, 2.0). In all three cases, an increase in λ_3 induces a transition from a one-way switch to a bistable switch, and to mono-stability. An increase in β leads to an increase in the size of both oneway and bistable regions in $\lambda_3 - G$ planes. It also moves up the bistable switch region. Therefore, the increased value of β creates richer dynamics for larger ranges of β_3: a mixture of a oneway switch, bistable switch and mono-stability of the core control system, thus, complex phenotypic switching between $\mathbb{T}_p$- and $\mathbb{T}_m$-types.

Sensitivity Analysis

In the model developed in this paper, there are a number of parameters for which no experimental data are known and/or they may have a significant effect on the results. We consider all parameters (G, S_1, S_2, λ_1, λ_2, λ_3, λ_4, λ_5, λ_6, α, β, γ, ϵ_1, ϵ_2) for sensitivity analysis of the core control model. In order to determine the sensitivity of the main variables (miR-451 level, AMPK activity, and mTOR activity) in the model at certain time to these parameters, we have performed sensitivity analysis. We have chosen a range for each of these parameters and divided each range into 10,000 intervals of uniform length. For each of the fourteen parameters of interest, a partial rank correlation coefficient (PRCC) value is calculated. PRCC values range between -1 and 1 with the sign determining whether an increase in the parameter value will decrease (-) or increase (+) the miR-451 level, AMPK activity, and mTOR activity at a given time. The sensitivity analysis to be described below was carried out using the method from [11] and Matlab files available from the website of Denise Kirschner's Lab: http://malthus.micro.med.umich.edu/lab/usadata/.

Fig S5 shows the sensitivity analysis results for the miR-451 level, AMPK activity, and mTOR activity at $t = 10$ (in (A)), 100 (in (B)). We calculate PRCC values and associated p-values for fourteen perturbed parameters (G, S_1, S_2, λ_g, λ_1, λ_2, λ_3, λ_4, λ_5, λ_6, α, β, γ, ϵ_1, ϵ_2) for the model (6)-(8), *i.e.,* we compute PRCC values for the miR-451 level, AMPK activity, and mTOR activity at time $t = 10, 100$. We conclude that the miR-451 level at $t = 100$ is positively correlated to the parameters G, λ_1, λ_2 but is poorly correlated (and thus not sensitive) to $S_2, \lambda_3, \lambda_4, \lambda_5, \lambda_6, \beta, \gamma, \epsilon_1, \epsilon_2$. Thus, in particular, the miR-

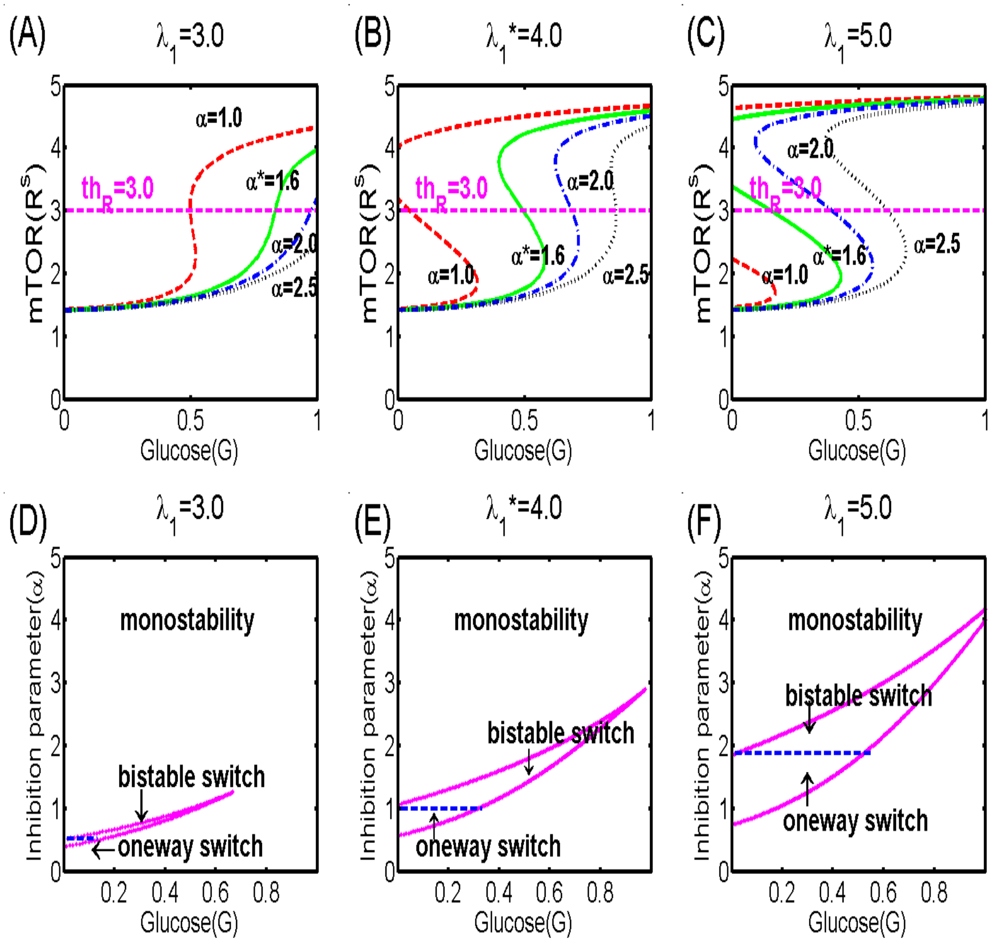

Figure S3. Bifurcation dynamics and bistable switches in response to various λ_1's. (A-C) Steady state values of mTOR activity (R^s) as a function of glucose level (G) for various inhibition strengths of miR-451 ($\alpha = 1.0, 1.6, 2.0, 2.5$) and for different autocatalytic parameters of miR-451 ($\lambda_1 = 3.0$ in (A), $\lambda_1 = 4.0$ in (B) (base value), $\lambda_1 = 5.0$ in (C)) in the core control system. Base values of α and λ_1 were marked with a star (*). (D-F) Diagram of bistable and oneway switches in a G-α plane when λ_1=3.0 (A), 4.0 (B), 5.0 (C). A magenta dotted line shows the boundary between oneway and bistable switches. Other parameters are fixed as in Table 1.

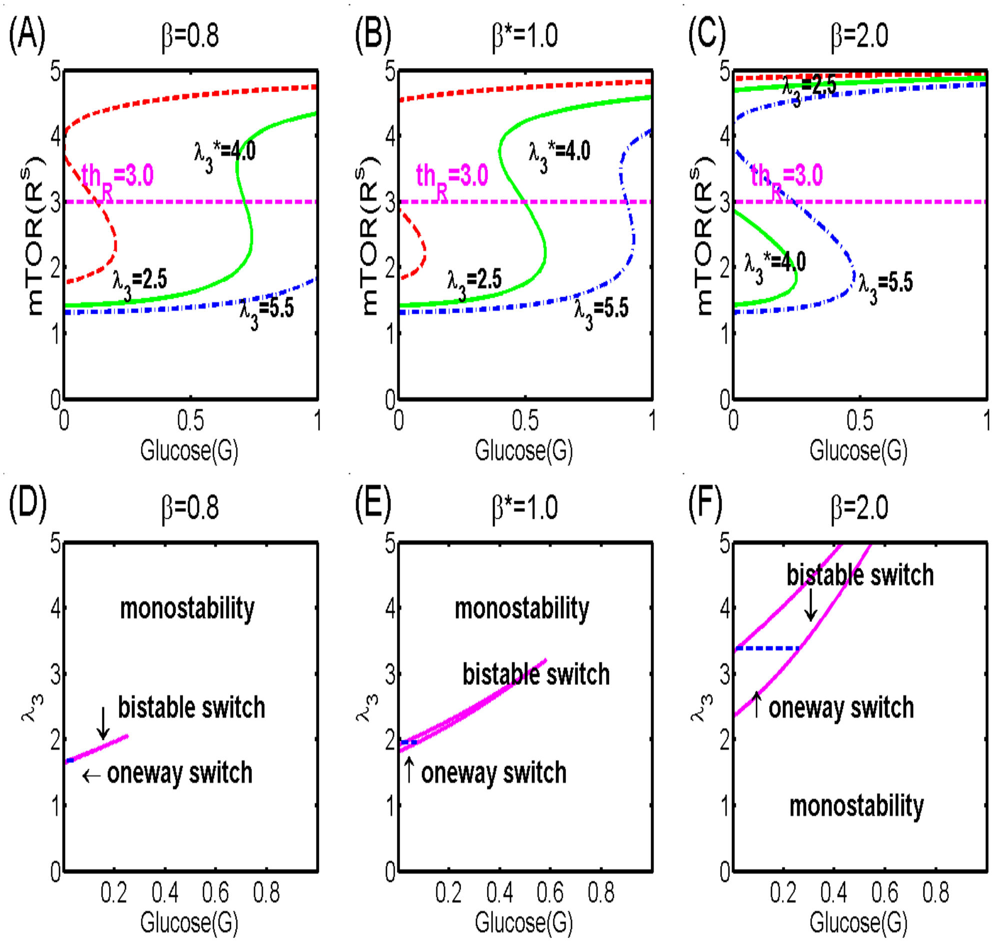

Figure S4. Bifurcation dynamics and bistable switches in response to various β's. (A) Steady state values of mTOR activity (R^s) as a function of glucose level (G) for various values of λ_3 ($\lambda_3 = 2.5, 4.0, 5.5$) when $\beta = 0.8$. (B) Same as in (A) but with $\beta = 1.0$. (C) Same as in (A) but with $\beta = 2.0$. (D-F) Diagram of bistable and oneway switches in G-λ_3 (=$[0, 1.3] \times [0, 8]$) plane for different βs ($\beta = 0.8, 1.0, 2.0$ in (D), (E), (F), respectively). The blue line illustrates the boundary ($\beta = 1.38$) between oneway and bistable switches. Base values of β and λ_3 were marked with a star (*). Other parameters are fixed as in Table 1.

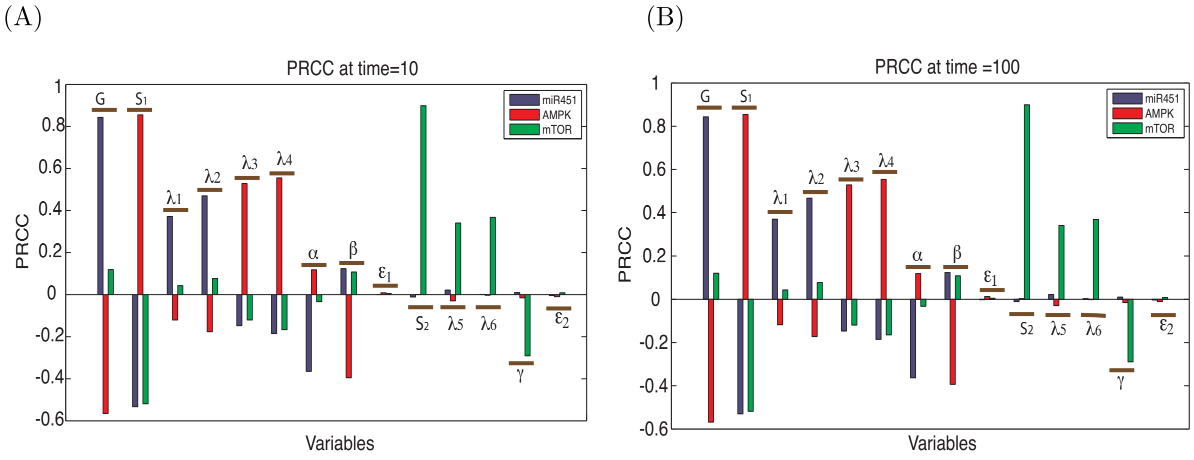

Figure S5. Sensitivity Analysis. General Latin Hypercube Sampling (LHS) scheme and Partial Rank Correlation Coefficient (PRCC) performed on the miR-451-AMPK-mTOR model. The y-value in subplots (A-B) indicates PRCC values of the miR-451 level, AMPK activity, and mTOR activity for model parameters $(G, S_1, S_2, \lambda_g, \lambda_1,\ \lambda_2,\ \lambda_3,\ \lambda_4,\ \lambda_5, \lambda_6, \alpha, \beta, \gamma, \epsilon_1, \epsilon_2)$ at $t = 10$ (in (A)), 100 (in (B)). The analysis was carried out using the method of [11] with sample size 10000.

451 activity will increase significantly if the glucose signal to miR-451 (G) and miR-451 autocatalytic production rates (λ_1, λ_2) are increased. On the other hand, the miR-451 activity is negatively correlated to the parameters S_1, α, *i.e.,* there would be significant decrease in the miR-451 level if the signal to the AMPK complex (S_1) and the inhibition strength of miR-451 by the AMPK complex (α) are increased. Due to mutual antagonism between the miR-451 level (M) and AMPK activity (A), it is expected to see opposite correlations between AMPK activity and those parameters. Indeed, AMPK activity is positively correlated to the parameters $S_1, \lambda_3, \lambda_4$ but negatively correlated to glucose signal (G) and inhibition parameter of AMPK (β). AMPK activity is not sensitive to parameters $\lambda_1, \lambda_2, \alpha, \epsilon_1$. Due to the structure of the model, it might be expected to see sensitivity of mTOR activity to parameters that are sensitive to miR-451 levels. However, mTOR activity is positively correlated to the parameters $(S_2, \lambda_5, \lambda_6)$ but is poorly correlated to $G, \lambda_1, \lambda_2, \lambda_3, \lambda_4, \alpha, \beta, \epsilon_1, \epsilon_2$. On the other hand, mTOR activity is negatively correlated to the signaling strength of the AMPK complex (S_1) and inhibition strength of mTOR by AMPK complex (γ). This is due to a strong inhibitory effect of LKB-AMPK on mTOR [1,12].

Therapeutic approaches: regulation of migration in glioblastoma

We test the effect of intervention of three pathways on regulation of cell migration and proliferation. Here, we assume that glucose is delivered to the glioma cell from blood vessels nearby, (ii) drugs can be injected either intravenously or via simple injection directly to the site. For this purpose, we introduce two equations for glucose (G) and drugs (D):

$$\frac{dG}{dt} = S_G \sum_{i=1}^{N_G} I_{[t_i, t_i + h_G]} - \mu_G G, \tag{10}$$

$$\frac{dD}{dt} = S_D \sum_{j=1}^{N_D} I_{[t_j, t_j + h_D]} - \mu_D D, \tag{11}$$

where μ_G is the consumption rate of glucose by brain tissue, μ_D is the natural decay rate of drugs. Here, S_G is the external periodic source of glucose from blood vessels on the time interval $[t_i, t_i + h_G],\ i = 1, \ldots, N_G$, for the time duration h_d and a period $\tau_G\ (= t_{i+1} - t_i,\ i = 1, \ldots, N_G - 1;\ h_G < \tau_G)$ between those intervals, N_G is the number of glucose injections, $I[\cdot]$ is the indicator function. In a similar fashion, S_D is the external periodic source of drugs on the time interval $[t_j, t_j + h_D],\ j = 1, \ldots, N_D$, for the time duration h_D and a period $\tau_D\ (= t_{j+1} - t_j,\ j = 1, \ldots, N_D - 1;\ h_D < \tau_D)$ between those intervals, N_D is the number of drug injections. In Figs S6A-S6F, we show the effect of inhibition of miR-451 by AMPK complex by a drug in Eq (11). So, we consider the modified equation

$$\epsilon_1 \frac{dA}{dt} = S_1 + \frac{\lambda_3 \lambda_4^2}{\lambda_4^2 + \beta e^{-\delta D} M^2} - A, \tag{12}$$

where δ is the effectiveness of the inhibitory drug on the pathway. Eqs (10)-(12) are coupled with the core control system (6)-(8) to see the effect of drugs in regulation of cell migration and proliferation. In Fig S6B we show dynamic regulation of cell proliferation and migration under various conditions in the $h_D - S_D$ space in response to fluctuating glucose in Fig S6D. Fig S6F shows time course of mTOR activities, in response to control (dotted), drug 1 (solid; $h_D = 0.5, S_D = 2$) and drug 2 (dashed; $h_D = 4.5, S_D = 2$) cases respectively. Fluctuating drug levels for those two cases are shown in Fig S6E. The system switches from the proliferative phase to the migratory phase when the injection duration of the drug (h_D) is increased (S_D is fixed) because of the weakened inhibition strength of AMPK complex activities. We conclude that the lower duration (h_D) and lower drug amount (S_D) induce the proliferative phase while cell migration takes place when either the duration (h_D) or drug amount (S_D) is increased. On the other hand, when we block the inhibition pathway of miR-451 by AMPK complex by replacing α with $\alpha e^{-\delta D}$, the system leads to the absolute proliferative phase in the same $h_D - S_D$ space (Fig S6A). The same effect of dominant proliferation modes can be obtained when we block the inhibition pathways of mTOR by AMPK complex, *i.e.,* $\gamma \to \gamma e^{-\delta D}$ (Fig S6C). These results indicate possible strategies of either increasing or decreasing cell migration at the molecular level. For instance, Kim *et al.* [7,13,14] suggested therapeutic strategies to use miR-451-AMPK core control system in terms of localization of invasive cells immediately after first surgery, followed by secondary surgery for removal of the localized invasive cells [15] on the periphery of original surgical site. An optimal control theory [16] was also applied for the strategy in order to obtain the optimal solution of maintaining 'non'-invasive status given glucose constraints since too much glucose could lead to clinical complications especially for diabetic patients with glioblastoma. AMPK stimulation or inhibition could lead to better clinical outcomes in different types and stages of cancer [12]. For instance, some authors reported that the stimulation of AMPK with pharmacological compounds may induce anti-tumoral effects in experiments [17,18] especially for diabetic type II patients with anti-diabetic drug metformin, toxic for malignant tumor cells [19]. However, activation of AMPK provides better chance of survival in many types of cancer. For instance, AMPK plays a central role in overcoming *anoikis* [20], programmed cell death under a loss of attachment to the basement membrane. AMPK is also the central coordinator of the metabolic reprogramming known as Warburg effect [21], a hallmark of malignant tumor cells, and AMPK inhibition rather than promotion may represent a potential therapeutic strategy in glioblastoma where the active infiltration of undetected cancer cells leads to final recurrence and poor clinical outcomes [22]. For example, compound C, a reversible and ATP-competitive AMPK inhibitor [23], was used to inhibit AMPK [24].

Glioma cells are exposed to highly heterogenous micro-environment such as fluctuating glucose levels. Experimentally it is not clear how many pathways or how fast action is necessary in order to induce the inhibitory action of miR-451 by AMPK complex. We consider two time delays in inhibition of pathways: (i) inhibition of miR-451 by AMPK complex (τ_1) (ii) inhibition of AMPK complex by miR-451 (τ_2). In Fig S7 we test the effect of time delays in the inhibition pathways on regulation of cell migration or

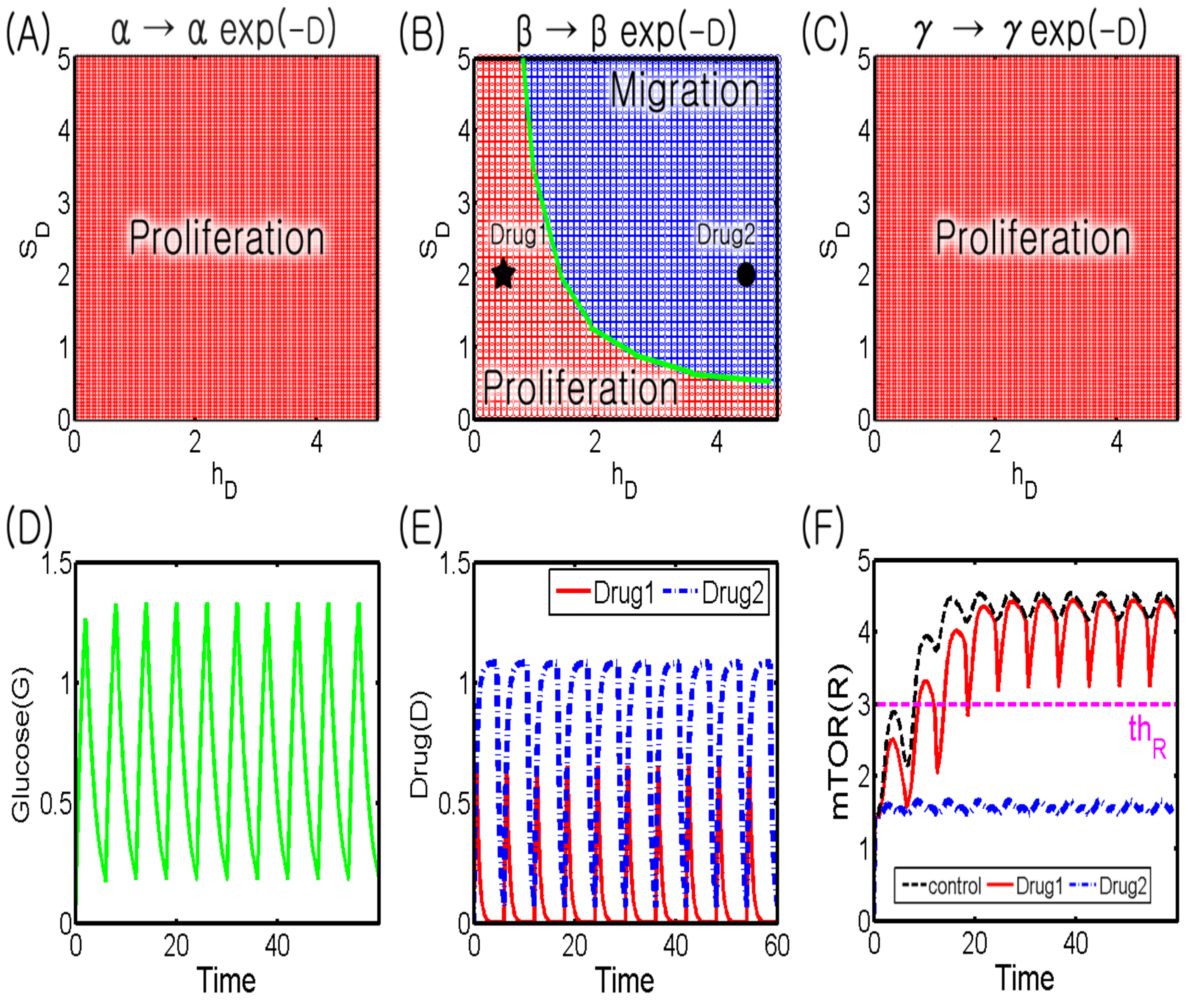

Figure S6. Effect of blocking signaling pathways on the dynamics of the core control system. (A-C) Dynamics of proliferation and migration in response to periodic injections of glucose and inhibitory drugs of miR-451 (A) AMPK complex (B) and mTOR (C) with durations h_G, h_D and injection strength S_G, S_D. The inhibitory pathways were blocked by replacing α, β, γ with αe^{-D}, βe^{-D}, γe^{-D}, respectively. Cell adapts to either proliferative (P) or migratory (M) phase in h_D-S_D parameter space (τ_D=1 fixed) at a final time point $t = 60$. (D-F) Time courses of glucose level (D), mTOR levels (F), and drugs (E) with the two parameter sets in (B) (star in (B) $h_D = 0.5, S_D = 2$ (solid line); circle in (B) $h_D = 4.5, S_D = 2$ (dashed line)). Initial conditions: $M(0)$=0.1, $A(0)$=4.0, $R(0) = 0.1$. Parameters: h_G=2.0, S_G=1.0, μ_G=0.5, τ_G=0.1, δ=1. Other parameters are same as in Table 1.

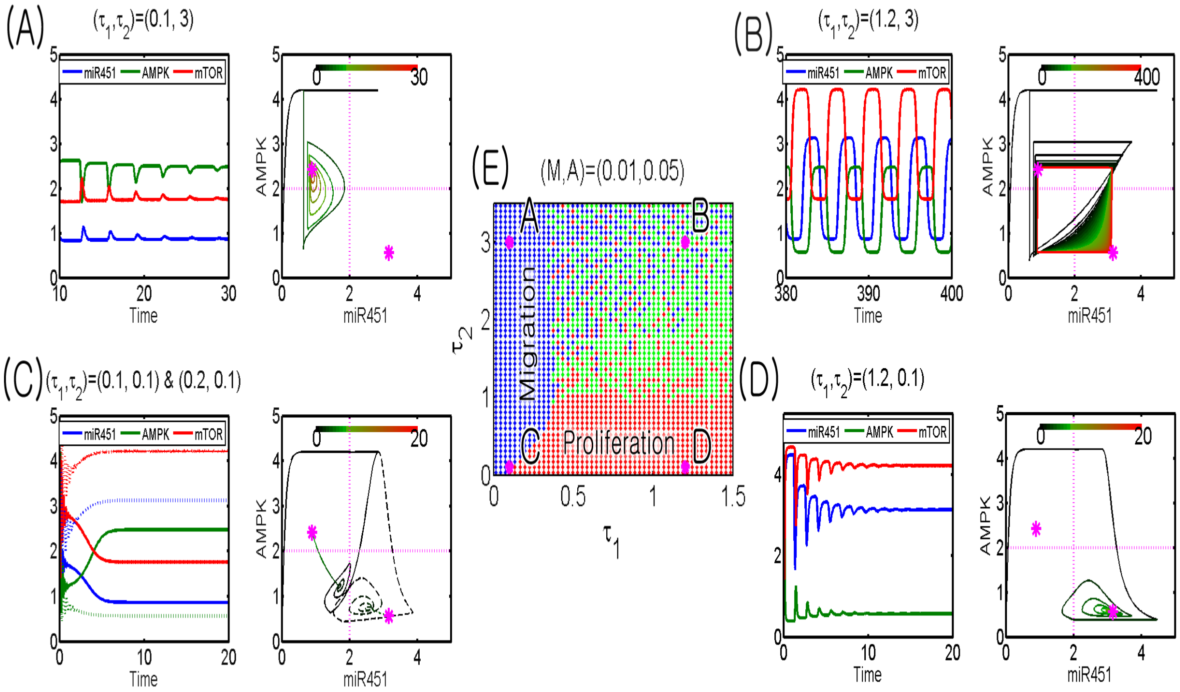

Figure S7. Effects of time delays on the dynamics of the core control system. Effects of time delays in inhibition pathways on regulation of proliferation and migration in response to an intermediate glucose level ($G = 0.5$). τ_1= time delay in inhibition of miR-451 by AMPK complex, τ_2= time delay in inhibition of AMPK complex by miR-451. (A-E) Characterization of two phenotypes in the $\tau_1 - \tau_2$ plane. Time courses of miR-451, AMPK, and mTOR (left panels) and trajectories on the $M - A$ plane (right panels) are shown in (A-D) for four points in (E): (τ_1, τ_2)=(0.1, 3) in (A), (1.2, 3) in (B), (0.1, 0.1) in (C), and (1.2, 0.1) in (D). Initial conditions: $M(0) = 0.01, A(0) = 0.05, R(0) = 4.0$. Two steady states were marked in star(*) in the right panels in (A-D).

proliferation. We use the following modified delay differential equations (DDEs)

$$\frac{dM}{dt} = \lambda_g G + \frac{\lambda_1 \lambda_2^2}{\lambda_2^2 + \alpha[A(t-\tau_1)]^2} - M, \tag{13}$$

$$\epsilon_1 \frac{dA}{dt} = S_1 + \frac{\lambda_3 \lambda_4^2}{\lambda_4^2 + \beta[M(t-\tau_2)]^2} - A, \tag{14}$$

$$\epsilon_2 \frac{dR}{dt} = S_2 + \frac{\lambda_5 \lambda_6^2}{\lambda_6^2 + \gamma A^2} - R, \tag{15}$$

where the time t was rescaled using $\tilde{t} = 10t$ for the computational convenience. While miR-451 directly acts on the AMPK complex, inhibition of miR-451 by AMPK complex involves indirect regulations via many other pathways [1], leading to delayed inhibition of AMPK complex by miR-451 (τ_2). Intervention of direct action on the AMPK complex by miR-451 will also induce time delays (τ_1). Different combinations of those two time delays in inhibition strengths lead to diverse behaviors in terms of cell migration and proliferation. In Fig S7 we investigate the effect of time delays on regulation of cell migration and proliferation in response to an intermediate glucose level ($G = 0.5$). The model predicts that tumor cells will adapt to the proliferative mode when $\tau_1 > \tau_2, \tau_2 < \tau_2^\dagger$ for a threshold $\tau_2^\dagger$ (Fig S7D) while the cells will switch to the migratory phase when $\tau_1 < \tau_2, \tau_1 < \tau_1^\dagger$ for a threshold $\tau_1^\dagger$ (Figure S7A). When the two time delays are small, the system leads to either proliferative or migratory phases depending on the relative strength of τ_1 and τ_2 (Fig S7C). Note that the cell is in the migratory phase for the same initial condition ($M(0) = 0.01, A(0) = 0.05, R(0) = 4.0$) in the absence of time delays, *i.e.,* when $\tau_1 = \tau_2 = 0$ (Fig 3). On the other hand, the system leads to the oscillations between $\mathbb{T}_p$ and $\mathbb{T}_m$ when τ_1, τ_2 are relatively large, for example, when $(\tau_1, \tau_2) = (1.2, 3)$ in Fig S7B. These results indicate the difficulty of treatment of GBM in the presence of those time delays (τ_1, τ_2) in a microenvironment where glucose may fluctuate, *i.e.,* possible drugs targeting AMPK inhibition may not work for heterogeneous glioma populations and other alternative strategies may need to be developed.

Stochastic fluctuations on signaling networks of miRNAs may play a significant role in regulation of cell fates [25]. In Fig S8 we illustrate the stochastic effect of the miR-451 and AMPK complex on the downstream mTOR activity, and regulation of cell migration in response to fluctuating glucose ($G(t) = 0.5 + 0.2\sin(\pi t/24)$) levels. Figs S8A-S8C show one realization for perturbed activities of miR-451 (Fig S8A) and AMPK complex (Fig S8B) in $G-M$ and $G-A$ planes, respectively, and time course of mTOR levels (Fig S8C). Gray S-curves in Figs S8A and S8B represent the bifurcation curve in the absence of noise (Fig 3J). While the system shows periodic oscillations in mTOR levels between $\mathbb{T}_p$ and $\mathbb{T}_m$ phases in the absence of noise (green solid line), it maintains the $\mathbb{T}_p$-phase in the first cycle (interval between arrows in Fig S8C) under the noise. Chances of infiltration of a glioma cell will be positively correlated to the time spent in the $\mathbb{T}_m$-phase, increasing the risk of tumor recurrence. Figs S8D and S8E show risk maps without (Fig S8D) and with (Fig S8E) noise for various combinations of α and β in response to fluctuating glucose and noise in Fig S8(A-C). While the overall pattern of high risk (smaller α and larger β) in Fig S8D is similar to the case with noise in Fig S8E, the intermediate boundary area between $\mathbb{T}_m$ and $\mathbb{T}_p$ is larger in Fig S8E. In other words, cell migration is more subject to stochastic noise in miR-451 and AMPK pathways. Finally, Fig S8F shows time courses of mTOR activities with the noise in the inhibition of mTOR activities by AMPK complex for various γ's ($\gamma = \gamma_0 + \sigma B(t)$; $\sigma = 0.5$, $\gamma_0 = 0.5$ (red), 1.0 (green), 2.0 (blue)). We found that the core control system is highly adaptive in terms of selecting the $\mathbb{T}_p$ and $\mathbb{T}_m$ phases even under this perturbation on the mTOR inhibition.

Discussion

miRNAs recently emerged as one of the key regulators of cellular process such as the cell cycle [8, 26]. Despite recent breakthroughs in our knowledge of these miRNAs, it is not clear yet how many miRNAs

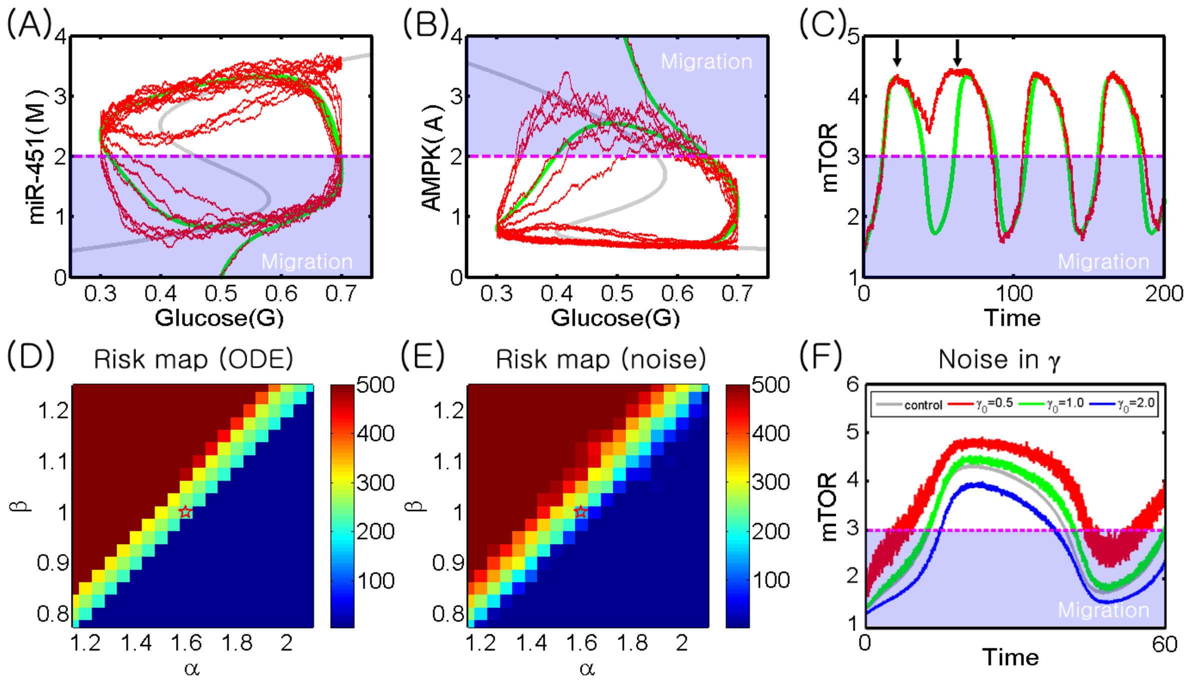

Figure S8. Stochastic effect on behaviors of the core system in response to fluctuating glucose levels ($G(t) = 0.5 + 0.2\sin(\pi t/24)$). The miR-451 and AMPK levels were perturbed by random Gaussian input (we added $\sigma dB(t)$ on the right-hand side of Eqs (6)-(7) where $B(t)$ is a standard Brownian motion). (A-C) One realization of the stochastic simulations for trajectories in $G - M$ (A) and $G - A$ planes (B), and the corresponding time course of mTOR activity (C) are shown in red. Gray bifurcation curves without noise are shown in (A,B). Green curves represent the trajectories (A,B) and time course (C) in the absence of noise. In response to glucose withdrawal, the system stays in the $\mathbb{T}_p$-phase with noise (between arrows) while it switches to the $\mathbb{T}_m$ phase; shaded light blue box) without noise (green curve). Other parameters are same as in Table 1. (D,E) Migration risk maps without (ODEs) and with noise (SDEs) on the $\beta - \alpha$ plane. The color bars indicate the amount of time spent in the dangerous migratory phase: $\tau^m = \int_0^T \chi_{E_M}(s)ds,\ E_M = \{t \in (0,T) : (M(t), A(t), R(t)) \in \mathbb{T}_m\}$. Here, $\chi_S(\cdot)$ is an characteristic function on a set S, which gives 1 on the set S and zero otherwise. Star in (D,E) indicates the base value of the parameters α, β. (F) One realization of stochastic simulations for time courses of mTOR levels for various inhibition strength of mTOR by AMPK complex (γ) in the presence of noise: $\gamma = \gamma_0 + \sigma B(t)$. Here γ_0 is the base value (γ_0=0.5 (red), 1.0 (green), 2.0 (blue) and σ is the perturbation strength. $\sigma = 0.5$. The curve without noise (control) was drawn in solid gray. Initial condition $M(0) = 0, A(0) = 4.2, R(0) = 1.4146$.

are involved in regulation of cancer progression or how these miRNAs exactly control the cellular process in tumor growth overall. Some upregulated miRNAs such as miR-21 in brain tumors are believed to play a pivotal role in pro-oncogenic activities supporting growth, proliferation, migration, and survival of cancer cells while expression of other miRNA is suppressed in gliomas. These miRNAs harbor a clinical significance as therapeutic components in anti-cancer therapy [27–31]. AMPK and mTOR are one of master players in metabolic reprogramming in glioma [32]. Godlewski *et al.* [1,33] identified a key molecular control system (miR-451, AMPK complex, and mTOR) that provides a critical switch between cell proliferation and migration. They [1,33] found that glucose can regulate glioma cell proliferation and migration via the core control system: (i) low glucose ⇒ down-regulation of miR-451, up-regulation of the AMPK complex, and down-regulation of mTOR ⇒ cell migration; (ii) normal (high) glucose ⇒ up-regulation of miR-451, down-regulation of AMPK complex, and up-regulation of mTOR ⇒ proliferation.

We developed a mathematical model of the core control system (miR-451-AMPK-mTOR) based on the experimental observations [1, 33] and analyzed the model's behavior in response to high and low glucose levels. The responses of the core control system for various glucose levels are in good agreement with experimental results [1,33]: In particular, (i) the up- and down-regulation of miR-451 and mTOR in response to high and low glucose levels, respectively, and (ii) the down- and up-regulation of the AMPK complex in response to normal and low glucose. This allowed us to define the migratory and proliferative phase based on the status of these molecules and the hysteresis system generates a bistable window (W_b in Fig 3J) as predicted in the previous smaller miR-451-AMPK model [6, 7]. The model system predicts oneway-, bistable-, and mono-stability switches under the perturbations of the key inhibition parameters α (Fig S1), β (Fig S2), γ (Fig S2)). Phenotypic changes under the perturbation of these parameters (α, β, γ) enabled us to predict the $\mathbb{T}_m/\mathbb{T}_p$-status of glioma cells in the glucose fluctuating microenvironment when specific components of the signaling system are perturbed, for example by miR-451-suppressing drugs (Figs S1,S2). The changes in the size and location of the bistable and one-way regions in $G-\alpha$ plane under perturbations of the inhibition (α) and enhancing (λ_1) parameters of miR-451 also generate a rich dynamics where the cell fate of migration and proliferation critically depends on the sign of dG/dt, a decrease in glucose (steady glucose withdrawal by the consumption) or increase in glucose (supply from blood vessels (BVs) or glucose intravenous infusion), and initial conditions (Fig S3). We also investigated the system responses under the perturbation of the inhibition (β) and enhancing (λ_3) parameter of the AMPK complex (Fig S4). Onset of $\mathbb{T}_p$ and $\mathbb{T}_m$ phenotypes in response to drugs targeting the inhibition pathways of the AMPK complex in the presence of fluctuating glucose are sensitivity to duration and strength of the drug while only $\mathbb{T}_p$-phenotype is selected for drugs targeting the inhibition pathways of miR-451 or mTOR (Fig S6). We also illustrated the difficulty of treating GBM in the presence of time delays (τ_1, τ_2) in the inhibition signaling network due to diverse responses of GBM cells (proliferation, migration, or periodic changes in $\mathbb{T}_p$ and $\mathbb{T}_m$ phases; Fig S7). In order to eradicate all heterogeneous glioma populations, alternative strategies may need to be developed. For instance, pre-conditioning chemotaxis-driven localization of cells [13, 14] after conventional surgery might be a way of homogenization of the tumor population in order to increase the efficacy of the drugs or clinical outcomes [34]. In this case, the optimized solutions need to be obtained in the controlling the core control system [16].

The growth pattern of a GBM can be determined based on perivascular accumulation of cancer cells, perineuronal satellitosis, subpial growth, and intrafascicular growth in the corpus callosum [22]. While miR-451 levels are up-regulated near blood vessels due to high levels of glucose supply [1,33], glucose levels and stiffness in brain tissue are irregular due to the heterogeneous microenvironment. Many of these microenvironmental factors in addition to intrinsic noise in vast signaling networks including the miR-45-AMPK-mTOR system may introduce biochemical perturbations within the core control system and the cell may select the proliferative phase instead of the migratory phase even under glucose conditions favoring migration (Fig S8). Cancer cells within tumors exist in distinct phenotypic states and stochasticity in individual behaviors may promote phenotypic equilibrium [35] and affect transitions between

$\mathbb{T}_m/\mathbb{T}_p$-status in glioblastoma, leading to heterogeneity in cancer populations [36].

References

1. Godlewski J, Nowicki MO, Bronisz A, Palatini GNJ, Lay MD, Brocklyn JV, et al. MircroRNA-451 regulates LKB1/AMPK signaling and allows adaptation to metabolic stress in glioma cells. Molecular Cell. 2010;37:620–632.

2. Khanin R, Vinciotti V. Computational modeling of post-transcriptional gene regulation by microRNAs. J Comput Biol. 2008;15(3):305–316.

3. Yao G, Lee TJ, Mori S, Nevins JR, You L. A bistable Rb-E2F switch underlies the restriction point. Nat Cell Biol. 2008;10(4):476–482.

4. Gantier MP, McCoy CE, Rusinova I, Saulep D, Wang D, Xu D, et al. Analysis of microRNA turnover in mammalian cells following Dicer1 ablation. Nucleic Acids Res. 2011;39(13):5692–703.

5. Crute BE, Seefeld K, Gamble J, Kemp BE, Witters LA. Functional domains of the alpha1 catalytic subunit of the AMP-activated protein kinase. J Biol Chem. 1998;273(52):35347–54.

6. Kim Y, Roh S, Lawler S, Friedman A. miR451 and AMPK/MARK mutual antagonism in glioma cells migration and proliferation. PLoS One. 2011;6(12):e28293.

7. Kim Y, Roh S. A hybrid model for cell proliferation and migration in glioblastoma. Discrete and Continuous Dynamical Systems-B. 2013;18(4):969–1015.

8. Aguda BD, Kim Y, Hunter MG, Friedman A, Marsh CB. MicroRNA Regulation of a Cancer Network: Consequences of the Feedback Loops Involving miR-17-92, E2F, and Myc. PNAS. 2008;105(50):19678–19683.

9. Ragan C, amd M A Ragan MZ. Quantitative prediction of miRNA-mRNA interaction based on equilibrium concentrations. PLoS Comput Biol. 2011;7(2):e1001090:doi:10.1371/journal.pcbi.1001090.

10. Hardie DG, Salt IP, Hawley SA, Davies SP. AMP-activated protein kinase : an ultrasensitive system for monitoring cellular energy charge. Biochem J. 1999;338:717–722.

11. Marino S, Hogue IB, Ray CJ, Kirschner DE. A methodology for performing global uncertainty and sensitivity analysis in systems biology. J Theor Biol. 2008;254(1):178–96.

12. Viollet B, Horman S, Leclerc J, Lantier L, Foretz M, Billaud M, et al. AMPK inhibition in health and disease. Crit Rev Biochem Mol Biol. 2010;45(4):276–95.

13. Kim Y. Regulation of cell proliferation and migration in glioblastoma: New therapeutic approach. Frontiers in Molecular and Cellular Oncology. 2013;3:53.

14. Kim Y, Powathil G, Kang H, Trucu D, Kim H, Lawler S, et al. Strategies of eradicating glioma cells: A multi-scale mathematical model with miR-451-AMPK-mTOR control. PLoS One. 2015;10(1):e0114370.

15. Juratli TA, Schackert G, Krex D. Current status of local therapy in malignant gliomas?a clinical review of three selected approaches. Pharmacology & therapeutics. 2013;139(3):341–358.

16. Schattler H, Kim Y, Ledzewicz U, d los Reyes V AA, Jung E. On the control of cell migration and proliferation in glioblastoma. Proceeding of the IEEE Conference on Decision and Control,. 2013;978-1-4673-5716-6/13:1810–1815.

17. Billaud M, Viollet B. Metformin in oncology, new clinical potential for an old remedy. Metformin: Mechanistic Insights Towards New Applications. 2008; p. 241–58.

18. Fogarty S, Hardie D. Development of protein kinase activators: AMPK as a target in metabolic disorders and cancer. Biochimica et Biophysica Acta (BBA)-Proteins and Proteomics. 2010;1804(3):581–591.

19. Buzzai M, Jones RG, Amaravadi RK, Lum JJ, DeBerardinis RJ, Zhao F, et al. Systemic treatment with the antidiabetic drug metformin selectively impairs p53-deficient tumor cell growth. Cancer research. 2007;67(14):6745–6752.

20. Schafer ZT, Grassian AR, Song L, Jiang Z, Gerhart-Hines Z, Irie HY, et al. Antioxidant and oncogene rescue of metabolic defects caused by loss of matrix attachment. Nature. 2009;461(7260):109–113.

21. Heiden MGV, Cantley LC, Thompson CB. Understanding the Warburg effect: the metabolic requirements of cell proliferation. Science. 2009;324(5930):1029–33.

22. Claes A, Idema AJ, Wesseling P. Diffuse glioma growth: a guerilla war. Acta Neuropathologica. 2007;114(5):443–458.

23. Zhou G, Myers R, Li Y, Chen Y, Shen X, Fenyk-Melody J, et al. Role of AMP-activated protein kinase in mechanism of metformin action. The Journal of clinical investigation. 2001;108(108 (8)):1167–1174.

24. Li J, Zeng Z, Viollet B, Ronnett GV, McCullough LD. Neuroprotective effects of adenosine monophosphate-activated protein kinase inhibition and gene deletion in stroke. Stroke. 2007;38(11):2992–2999.

25. Giampieri E, Remondini D, de Oliveira L, Castellania G, Lio P. Stochastic analysis of a miRNA-protein toggle switch. Mol BioSyst. 2011;7:2796–2803.

26. Mens MMJ, Ghanbari M. Cell Cycle Regulation of Stem Cells by MicroRNAs. Stem Cell Rev. 2018;14(3):309–322.

27. Hosseinahli N, Aghapour M, Duijf PHG, Baradaran B. Treating cancer with microRNA replacement therapy: A literature review. J Cell Physiol. 2018;233(8):5574–5588.

28. Rupaimoole R, Slack FJ. MicroRNA therapeutics: towards a new era for the management of cancer and other diseases. Nat Rev Drug Discov. 2017;16(3):203–222.

29. Chistiakov DA, Chekhonin VP. Contribution of microRNAs to radio- and chemoresistance of brain tumors and their therapeutic potential. Eur J Pharmacol. 2012;684(1-3):8–18.

30. Godlewski J, Newton HB, Chiocca EA, Lawler SE. MicroRNAs and glioblastoma; the stem cell connection. Cell Death Differ. 2010;17(2):221–8.

31. Lawler S, Chiocca EA. Emerging functions of microRNAs in glioblastoma. J Neurooncol. 2009;92(3):297–306.

32. Strickland M, Stoll EA. Metabolic Reprogramming in Glioma. Front Cell Dev Biol. 2017;5:43.

33. Godlewski J, Bronisz A, Nowicki MO, Chiocca EA, Lawler S. microRNA-451: A conditional switch controlling glioma cell proliferation and migration. Cell Cycle. 2010;9(14):2742–8.

34. Watts C, Price S, Santarius T. Current Concepts in the Surgical Management of Glioma Patients. Clinical Oncology. 2014;.

35. Gupta PB, Fillmore CM, Jiang G, Shapira SD, Tao K, Kuperwasser C, et al. Stochastic state transitions give rise to phenotypic equilibrium in populations of cancer cells. Cell. 2011;146(4):633–644.

36. Katsushima K, Kondo Y. Non-coding RNAs as epigenetic regulator of glioma stem-like cell differentiation. Front Genet. 2014;5:14.
